# Upregulation of GPNCA is associated with poor prognosis through enhancement of tumor growth via regulating GSK3B

**DOI:** 10.1038/s41598-020-58729-6

**Published:** 2020-02-06

**Authors:** Weijie Liao, Fuhai Liu, Haowei Zhang, Weifang Liao, Naihan Xu, Weidong Xie, Yaou Zhang

**Affiliations:** 1 School of Life Sciences, Tsinghua University, Beijing, 100084 P.R. China; 2 Key Lab in Healthy Science and Technology, Division of Life Science, Graduate School at Shenzhen, Tsinghua University, Shenzhen, 518055 P.R. China; 3 State Key Laboratory of Chemical Oncogenomic, Graduate School at Shenzhen, Tsinghua University, Shenzhen, P.R. China; 4 Open FIESTA Center, Tsinghua University, Shenzhen, 518055 P.R. China; 5 School of biology and pharmaceutical engineering, Wuhan Polytechnic University, Wuhan, 430023 P.R. China

**Keywords:** Targeted therapies, Diagnostic markers

## Abstract

GPNCA is a long non-coding RNA with unknown functions. In this study, using data from 9 cancers obtained from The Cancer Genome Atlas (TCGA), GPNCA was identified as overexpressed in cancer vs. normal tissues. The upregulation of GPNCA was associated with poor overall prognosis in colon, liver, renal clear cell and breast cancers. The upregulation of GPNCA was partly due to enhanced H3K27ac occupancy on its promoter region via EP300 and KAT2A/GCN5. The overexpression of GPNCA was positively related to tumor metastasis in colon cancer and poor disease-free and recurrence-free survival in colon and liver cancer. Both gene ontology (GO) enrichment and Kyoto encyclopedia of genes and genomes (KEGG) pathway enrichment analysis indicated that GPNCA was closely linked to regulation of gene transcription and post-transcriptional modifications, which was further supported by *in vitro* cell cytoplasmic and nuclear RNA purification assessments. Furthermore, GPNCA was associated with cell growth. Our *in vitro* experiments demonstrated that GPNCA silencing inhibited tumor growth via inhibiting its nearby gene GSK3B. Taken together, these findings highlight GPNCA as a biomarker for cancer diagnosis and a potential target for future cancer drug development.

## Introduction

Cancer is now the 2nd most fatal non-communicable disease globally, following only cardio cerebrovascular disease^1^. Amongst the life-threatening cancers, liver and colorectal cancers rank in the top 10 for both incidence and mortality^2^. The major characteristics of cancer cells are their ability to compete with normal cells for nutritional intake, coupled to unregulated cell growth and proliferation^3^. One of the main causes of death for cancer patients is delayed diagnosis. Gene-expression signatures hold great value as diagnostic and prognostic biomarkers^4^. Despite recent diagnostics moving towards molecular therapies, progress in this area remains modest. New and accurate cancer biomarkers are therefore urgently needed^5^.

Non-coding RNAs are novel biological molecules that were previously considered junk genes^6,7^. Among these, long non-coding RNAs (LncRNAs) are transcripts larger than 200 nucleotides that lack protein-coding ability or possess small peptide coding capacity^8–10^. During recent years, studies have identified an array of cellular functions associated with LncRNAs, including chromatin modification, gene transcription and posttranscriptional regulation^11,12^. In many of these studies, lncRNAs were shown to be abnormally expressed in many cancers, contributing to tumorigenesis and tumor progression. For example, lncRNA HOTAIR increased cancer invasiveness and metastasis in an PRC2-dependent manner^13^; LincRNA ZEB1-AS1 is up-regulated in hepatocellular carcinoma (HCC) and promotes tumor growth and metastasis through targeting ZEB1^14^; LncRNA HULC up-regulates HMGA2 as a microRNA sponge and promotes liver cancer growth^15^; LncRNA HNF1A-AS1 is upregulated in colon cancer and mediates metastatic progression in part through the miR34a/p53 signaling axis^16^.

GPNCA is a novel lncRNA located in the 3q13.33 (120094895–120136783) region with unknown cellular functions. To reveal new functions for GPNCA, we searched RNA-seq data from nine cancers in The Cancer Genome Atlas (TCGA) database and observed its up-regulation in each cancer-type, which in turn was associated with poor overall survival in colon cancer, liver cancer, breast cancer and renal clear cell carcinoma. GO and KEGG enrichment analysis revealed that GPNCA participated in an array of biological processes and pathways associated with tumor development. Finally, we confirmed that GPNCA silencing could inhibit HepG2 and HCT116 tumor cell growth via regulating its nearby gene GSK3B. Taken together, these data demonstrate that the high expression of LncRNA GPNCA is responsible for the cancer incidence and poor overall survival, and drugs targeting GPNCA may represent a potential effective treatment for colon and liver cancer.

## Results

### Discovery and partly overview of GPNCA

**Figure 1:**
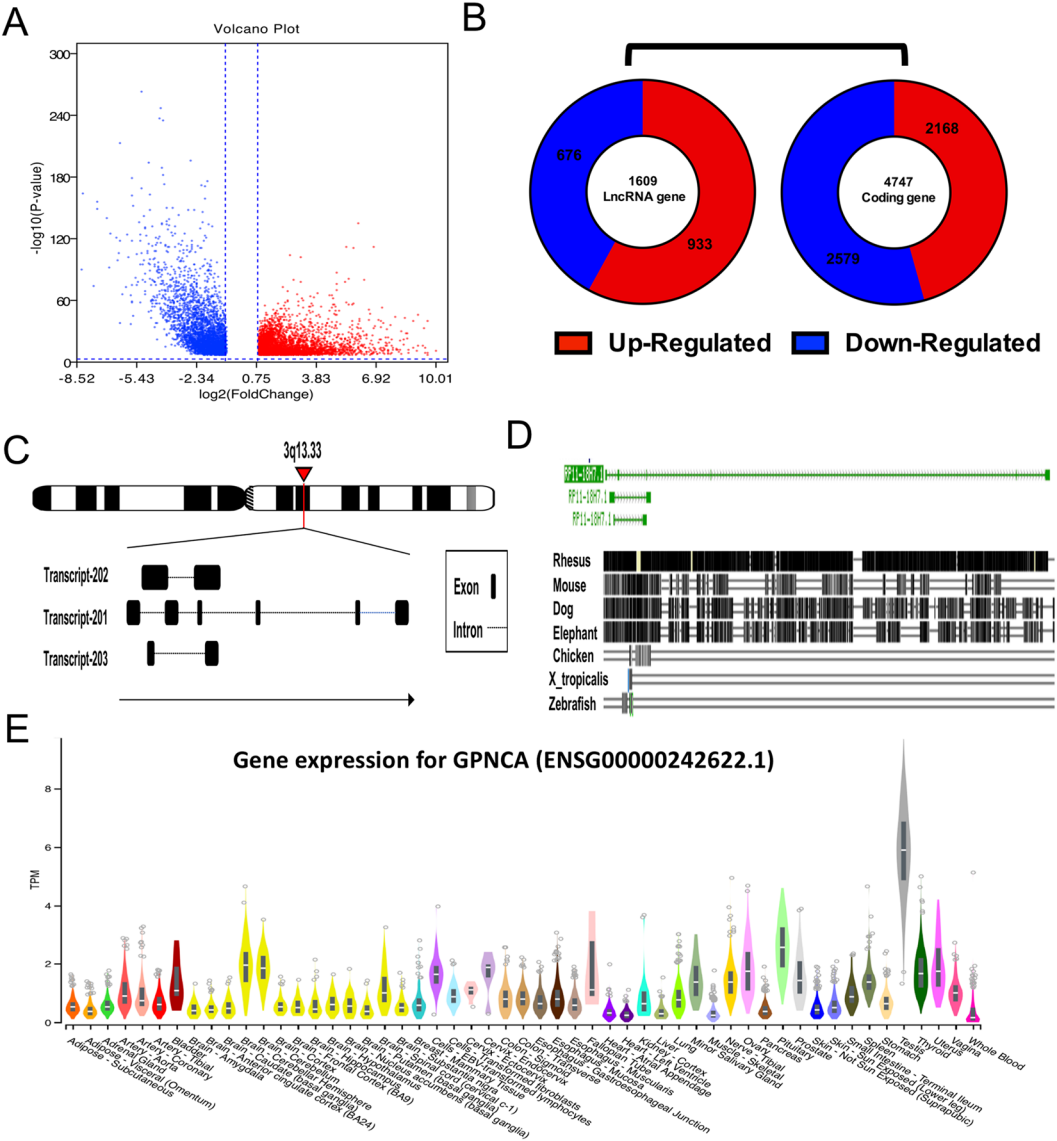
Discovery and overview of GPNCA. (**A**) Gene differential expression analysis between colon cancer (n = 480) and normal tissue (n = 41) using RNA-seq counts from the TCGA database, and cut-off values set as absolute *log2 (FC) values* > *0.85, p* < *0.001*. (**B**) Statistics for coding genes (n = 4747) and lncRNA genes (n = 1609) for A. (**C**) Diagrams of location and transcripts of GPNCA obtained from *Ensembl*. (**D**) Six of the GPNCA orthologues from 100 vertebrate species were monitored via the Multiz Alignments track in the UCSC Genome Browser. (**E**) Expression of EPB41L4A-AS1 provided by RNA-Seq data from GTEx (53 Tissues, 570 Donors) in the GTEXPORTAL.

LncRNAs are dysregulated in an array of cancers and contribute to disease progression. To discover new lncRNAs related to colon cancer progression, differential gene expression analysis was performed between normal and tumor tissues for over 7000 genes (Fig. 1A). Amongst the 1609 abnormally expressed lncRNAs identified, 933 were upregulated and 676 were down-regulated (Fig. 1B). Here, we termed LncRNA GPNCA whose gene *Ensembl ID* was ENSG00000242622.1 and was upregulated in the Chr3q13.33 (120094895-120136783) region next to GSK3B. According to the *Ensembl* database, three transcripts with 982 bp, 969 bp and 545 bp respectively were identified (Fig. 1C). The lncRNAs typically showed low homology and tissue-specificity across different species. According to the UCSC database, LncRNA GPNCA showed high homology to Rhesus but little homology to Mouse, Dog, Elephant, Chicken, X_tropicalis and Zebrafish (Fig. 1D). LncRNA GPNCA showed no tissue-specificity and ubiquitously expressed (Fig. 1E).

### LncRNA GPNCA expression is up-regulated in different solid tumors

**Figure 2:**
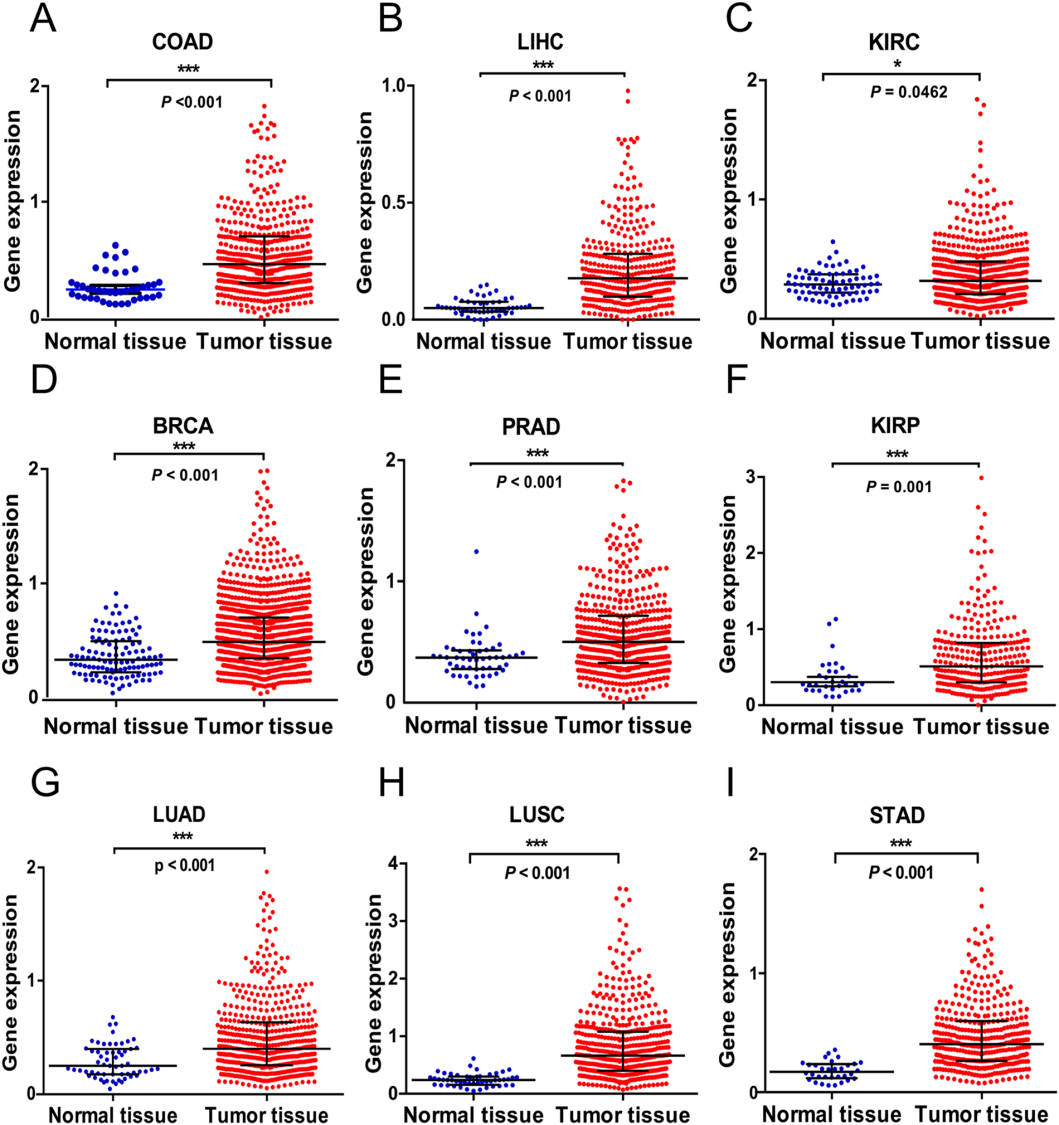
GPNCA is up-regulated in different solid tumors. Expression of GPNCA in TCGA: (**A**) Colon adenocarcinoma (COAD) tumor tissue (n = 480) and normal tissue (n = 41), (**B**) Liver hepatocellular carcinoma (LIHC) tumor tissue (n = 374) and normal tissue (n = 50), (**C**) Kidney renal clear cell carcinoma (KIRC) tumor tissue (n = 538) and normal tissue (n = 72), (**D**) Breast invasive carcinoma (BRCA) tumor tissue (n = 1102) and normal tissue (n = 113), (**E**) Prostate adenocarcinoma (PRAD) tumor tissue (n = 499) and normal tissue (n = 52), (**F**) Kidney renal papillary cell KIRP carcinoma (KIRP) tumor tissue (n = 288) and normal tissue (n = 32), (**G**) Lung adenocarcinoma (LUAD) tumor tissue (n = 533) and normal tissue (n = 59), (**H**) Lung squamous cell carcinoma (LUSC) tumor tissue (n = 502) and normal tissue (n = 49), (**I**) Stomach adenocarcinoma (STAD) tumor tissue (n = 375) and normal tissue (n = 32). Data were showed as median with interquartile range, *p < 0.05, **p < 0.01, ***p < *0.001*.

To evaluate the upregulation of lncRNA GPNCA in patients with different solid tumors, RNA-seq data from the *TCGA database* were analyzed. The results showed that GPNCA was notably overexpressed in colon cancer tissues (COAD, *p* < *0.001*; Fig. 2A), liver cancer tissue (LIHC, *p* < *0.001*; Fig. 2B), renal clear cell cancer tissue (KIRC, *p* = *0.046*; Fig. 2C), breast cancer tissue (BRCA, *p* < *0.001*; Fig. 2D), prostate cancer tissue (PRAD, *p* < *0.001*; Fig. 2E), Kidney renal papillary cell cancer tissue (KIRP, *p* < *0.001*; Fig. 2F), lung adenocarcinoma tissue (LUAD, *p* < *0.001*; Fig. 2G), lung squamous cell carcinoma tissue (LUSC, *p* < *0.001*; Fig. 2H) and stomach adenocarcinoma tissue (STAD, *p* < *0.001*; Fig. 2I), compared to each normal tissue. These results suggested that GPNCA had consistent expression characteristics across a range of solid tumors, indicating its potential as an oncogene and diagnostic biomarker for these important cancer-types.

### High expression of lncRNA GPNCA is associated with poor survival

**Figure 3:**
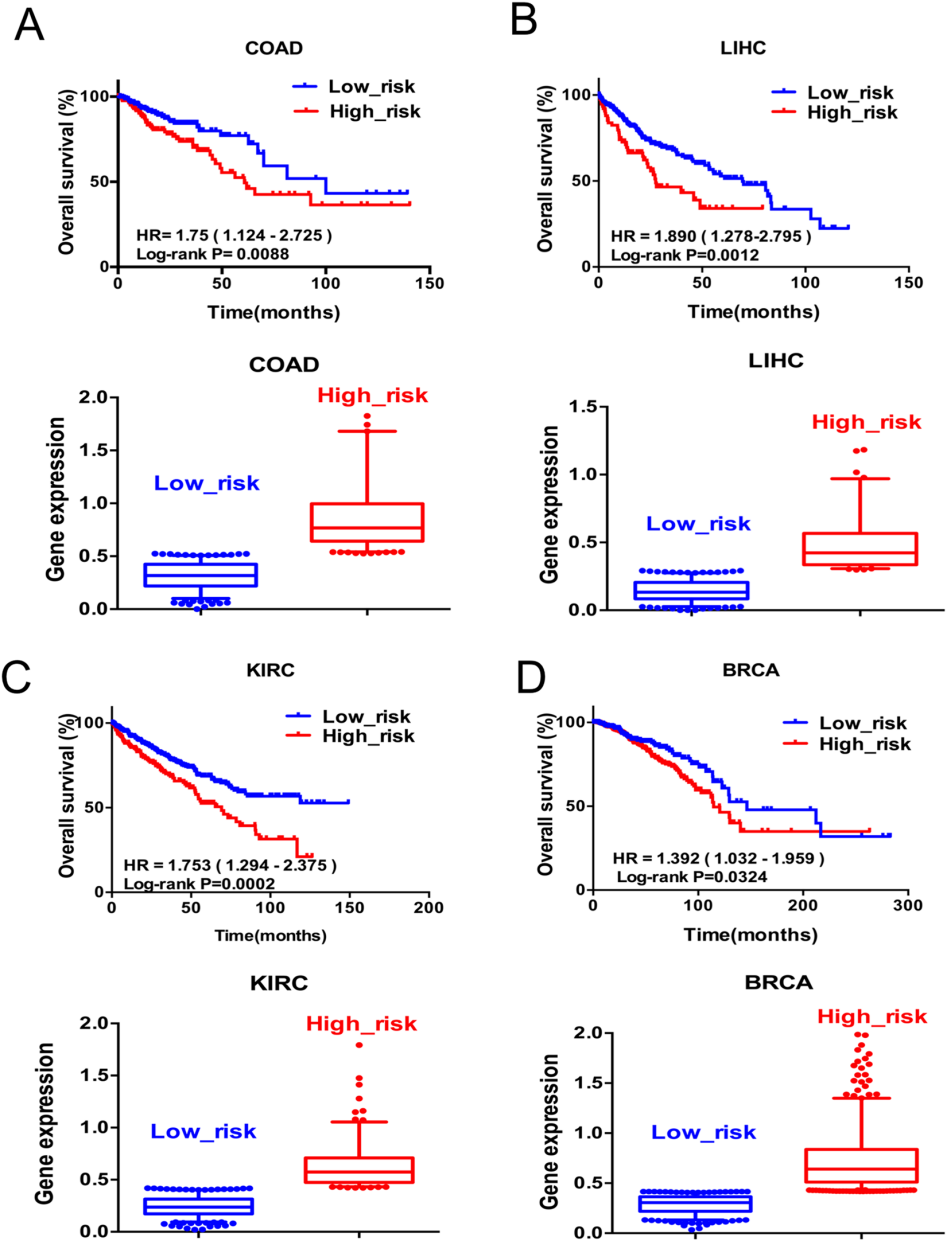
High expression of GPNCA was associated with poor OS. Risk analyses from tumor RNA-seq FPKM data and clinical data from (**A**) colon adenocarcinoma (lower, n = 480), (**B**) Liver hepatocellular carcinoma (lower, n = 370), (**C**) Kidney renal clear cell carcinoma (lower, n = 530), (**D**) Breast invasive carcinoma (lower, n = 1102). Low risk is shown as blue and high risk are shown as red. Kaplan-Meier survival curves of Cox analysis for (**A**) colon adenocarcinoma (upper), (**B**) Liver hepatocellular carcinoma (upper), (**C**) Kidney renal clear cell carcinoma (upper), (**D**) Breast invasive carcinoma (upper).

To investigate the relationship between lncRNA GPNCA and prognosis in colon cancer patients, RNA-seq data and clinicopathologic information of colon cancer patients from the *TCGA* were used for analysis. Firstly, receiver operating characteristic curve (ROC) was constructed to stratify patients into two subgroups according to GPNCA expression, FPKM values and patients’ overall status. Kaplan-Meier survival curves were plotted to compare OS between the subgroups. Colon cancer patients with LncRNA GPNCA expression (FPKM value) ≤ 0.525 were divided into low-risk groups while those ≥0.525 were divided into high-risk groups. High-risk patients showed poor OS in colon cancer (HR = 1.75, 95% CI, 1.124–2.725, p = 0.0088) (Fig. 3A and Supplemental Fig. S1A). Moreover, we also analyzed the OS of liver cancer patients, renal clear cell cancer patients and breast cancer patients. The results from liver cancer patients showed that those with GPNCA expression (FPKM value) > 0.295 were of high-risk and had poor survival (HR = 1.890, 95% CI, 1.278–2.795, P = 0.0012), (Fig. 3B and Supplemental Fig. S1B); whilst renal clear cell cancer patients with GPNCA expression (FPKM value) > 0.421 were of high-risk resulting in poor OS (AUC value > 0.5, HR = 2.166, 95% CI, 1.269–3.698, P = 0.0002)(Fig. 3C and Supplemental Fig. S1C). The results of breast cancer patients with GPNCA expression (FPKM value) ≥ 0.417 were of high-risk leading to poor OS (HR = 1.392, 95% CI, 1.032–1.959, P = 0.0324) (Fig. 3D and Supplemental Fig. S1D). These data suggested that high GPNCA expression was indeed associated with poor OS in several cancers but not enough sensitive to be an independent clinical biomarker for prognosis.

### Abnormal expression of lncRNA GPNCA influences colon and liver cancer progression

**Figure 4:**
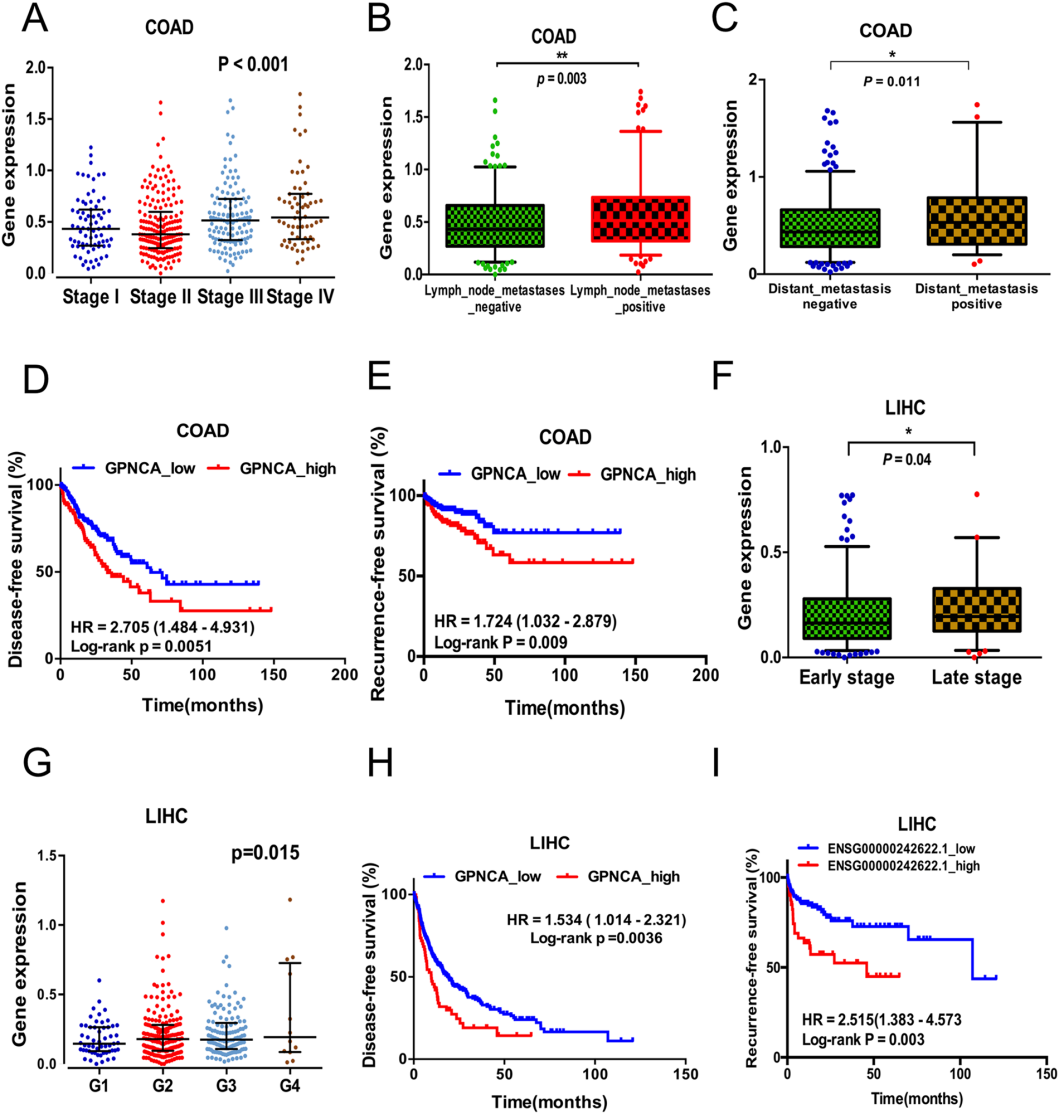
Abnormal expression of lncRNA GPNCA in colon and liver cancer. (**A**) GPNCA expression in different neoplasm disease stages of colon adenocarcinoma (Stage I, n = 75; Stage II, n = 176; Stage III, n = 128; Stage IV, n = 64), *one-way ANOVA*. (**B**) Expression of GPNCA in lymph node metastases positive (n = 171) or negative (n = 243) colon adenocarcinoma. (**C**) Analysis of GPCNA expression in distant metastasis negative (n = 308) and positive (n = 54) colon adenocarcinoma. Kaplan-Meier survival curves between GPNCA expression and (**D**) DFS (n = 455) and (**E**) RFS (n = 365) for colon adenocarcinoma. (**F**) GCPNA expression in early neoplasm disease stages (Stage I + Stage II, n = 255) and late neoplasm disease stages (Stage III + Stage IV, n = 84) for liver hepatocellular carcinoma. (**G**) Analysis of GPNCA expression in different differentiated grade tissues (G1 = 55, G2 = 177, G3 = 122, G4 = 12) of liver HCC. Kaplan-Meier survival curves between GPNCA expression and (**H**) disease-free survival (n = 364) or (**I**) recurrence-free survival (n = 190) for liver hepatocellular adenocarcinoma. *p < 0.05, **p < 0.01, ***p < *0.001*.

To further investigate the relationship between lncRNA GPNCA and the occurrence and progression of colon and liver cancer, the clinicopathologic information were analyzed in more detail. We found that GPNCA was markedly upregulated in colon cancer according to neoplasm disease (*p* < *0.001*; Fig. 4A). Moreover, patients with colon cancer who were lymph node metastases positive (*p* = *0.00*2*9*; Fig. 4B) and distant metastasis positive (*p* = *0.011*; Fig. 4C) showed higher expression of GPNCA. In addition, GPNCA patients showed poor DFS (HR = 1.498, 95% CI, 1.072–2.094, *P* = *0.01*) (Fig. 4D) and poor RFS (HR = 1.724, 95% CI, 1.032–2.879, *p* = *0.009*) (Fig. 4E). We next confirmed these findings using RNA-seq and clinicopathologic information in liver cancer patients. As data was lacking for neoplasm disease stage IV patients, we subdivided individuals into early stages containing stage I and stage II, and late stages containing stage III and stage IV. Higher levels of lncRNA GPNCA expression were observed in patients with late stage disease (P = 0.04, Fig. 4F). These data further indicated that GPNCA is intricately linked to colon and liver cancer progression. In addition, the upregulation of GPNCA was more obvious in poorly differentiated liver cancer tumor tissue (*p* = *0.015*; Fig. 4G). Liver cancer patients with high levels of GPNCA expression also showed poor DFS (HR = 1.493, 95% CI, 1.068–2.088, *p* = *0.01*) (Fig. 4H) and RFS (HR = 2.515, 95% CI, 1.383–4.573, *p* = *0.003*) (Fig. 4I).

### GPNCA expression is regulated by histone 3 acetylation around its promoter region in liver cancer

**Figure 5:**
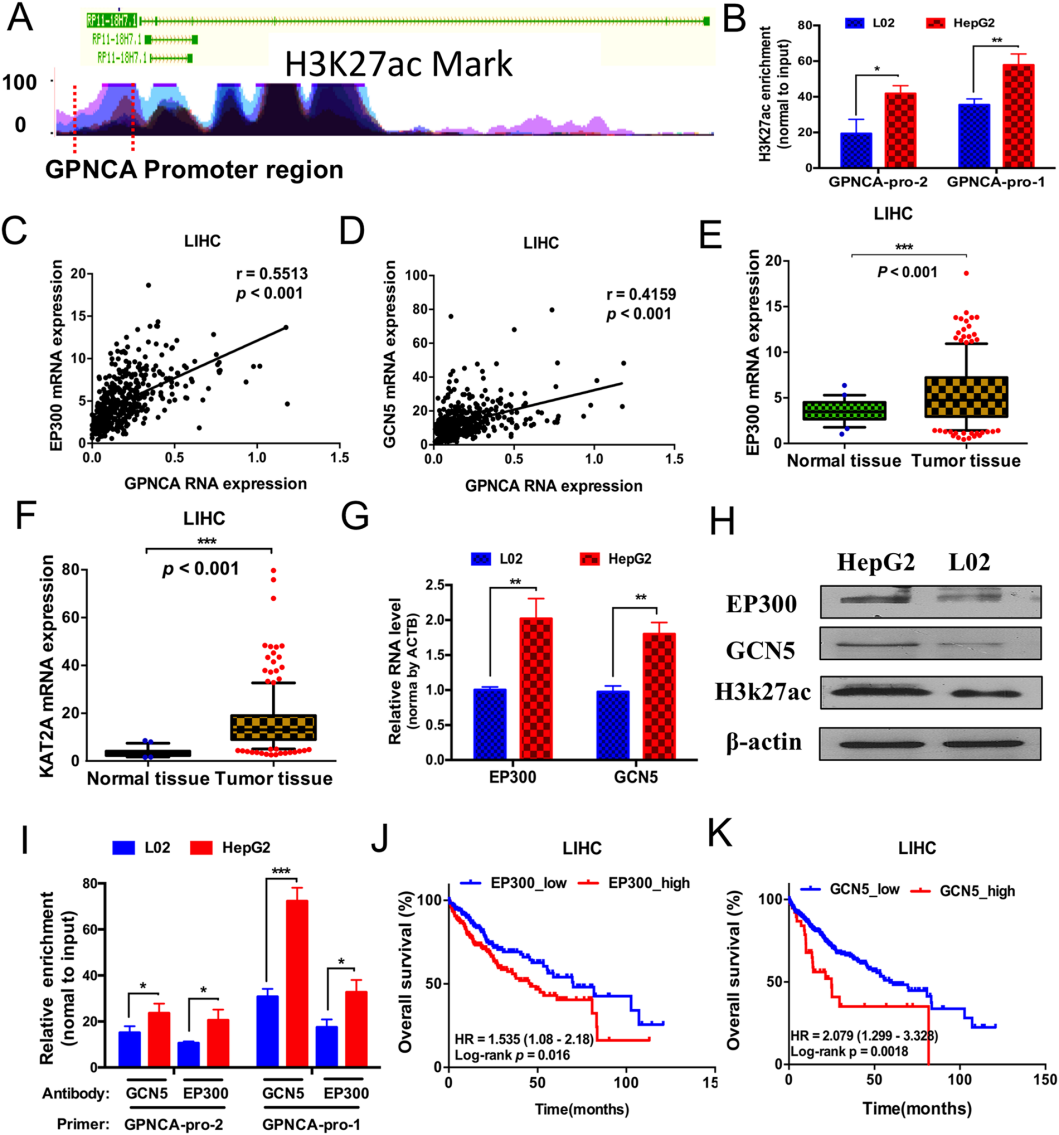
Acetylation levels of histone 3 regulated GPNCA expression. (**A**) H3k27ac enrichment across the GPNCA gene determined by ChIP-seq from the UCSC genome browser. The region between the two red dotted lines represents the promoter region. (**B**) ChIP assays were used to measure H3k27ac enrichment around the GPNCA promoter in HepG2 and L02 cells (n = 9). (**C**,**D**) Gene correlation analysis between GPNCA and EP300 (n = 424) or GCN5 (n = 424), using the FPKM values of liver cancer data from TCGA. (**E**,**F**) EP300 expression in normal liver tissue (n = 50) and liver tumor tissue (n = 374), GCN5 expression in normal liver tissue (n = 50) and liver tumor tissue (n = 374). Data were obtained from the TCGA. (**G**,**H**) mRNA expression (n = 9) or protein levels of EP300 and GCN5 in HepG2 and L02 cells measured by qRT-PCR or western blot. (**I**) EP300 and GCN5 occupation on the GPNCA gene promoter region were detected by ChIP assays in HepG2 and L02 cells (n = 9). (**J**,**K**) Kaplan-Meier survival curves of Cox analysis for EP300 (n = 370) and GCN5 (n = 370) in liver cancer patients. *p < 0.05, **p < 0.01, ***p < *0.001*.

DNA methylation and histone modifications have long been used to study abnormal genome transcription during cancer development. Herein, we investigated histone modification around the GPNCA promoter and identified an enrichment of H3k27ac, which positively correlated with gene expression. (Fig. 5A). *In intro* ChIP (Chromatin immunoprecipitation) assays revealed that H3k27ac enrichment was markedly higher in HepG2 cells (liver cancer cells) compared to LO2 cells (normal liver cells) on the GPNCA promoter region (Fig. 5B). Since histone acetylation is regulated by acetyltransferase, gene correlation analysis was performed to verify the correlation between GPNCA and acetyltransferase genes with liver cancer data from the TCGA. The Pearson’s correlation coefficient of EP300 and GPNCA was 0.5513 (*p* < *0.001*) while the correlation coefficient of GCN5 and GPNCA was 0.4159 (*p* < *0.001*), which showed positive correlation to GPNCA (Fig. 5C,D). EP300 (*p* < 0.001) and GCN5 (*p* < 0.001) were highly expressed in liver tumor tissue compared to normal tissue (Fig. 5E,F) which was confirmed by *in vitro* qPCR assays and western blot analysis (Fig. 5G,H, Supplemental Fig. S2). EP300 and GCN5 showed higher occupancy on the GPNCA promoter region in liver cancer cells demonstrated by ChIP assays (Fig. 5I). In addition, the high expression of EP300 (HR = 1.535, 95% CI, 1.08–2.18, *p* = *0.016*) and GCN5 (HR = 2.079, 95% CI, 1.299–3.328, *P* = *0.0018*) were both associated with poor OS (Fig. 5J,K).

### LncRNA GPNCA and its co-expressed genes participate in tumor associated biological processes and pathways

**Figure 6:**
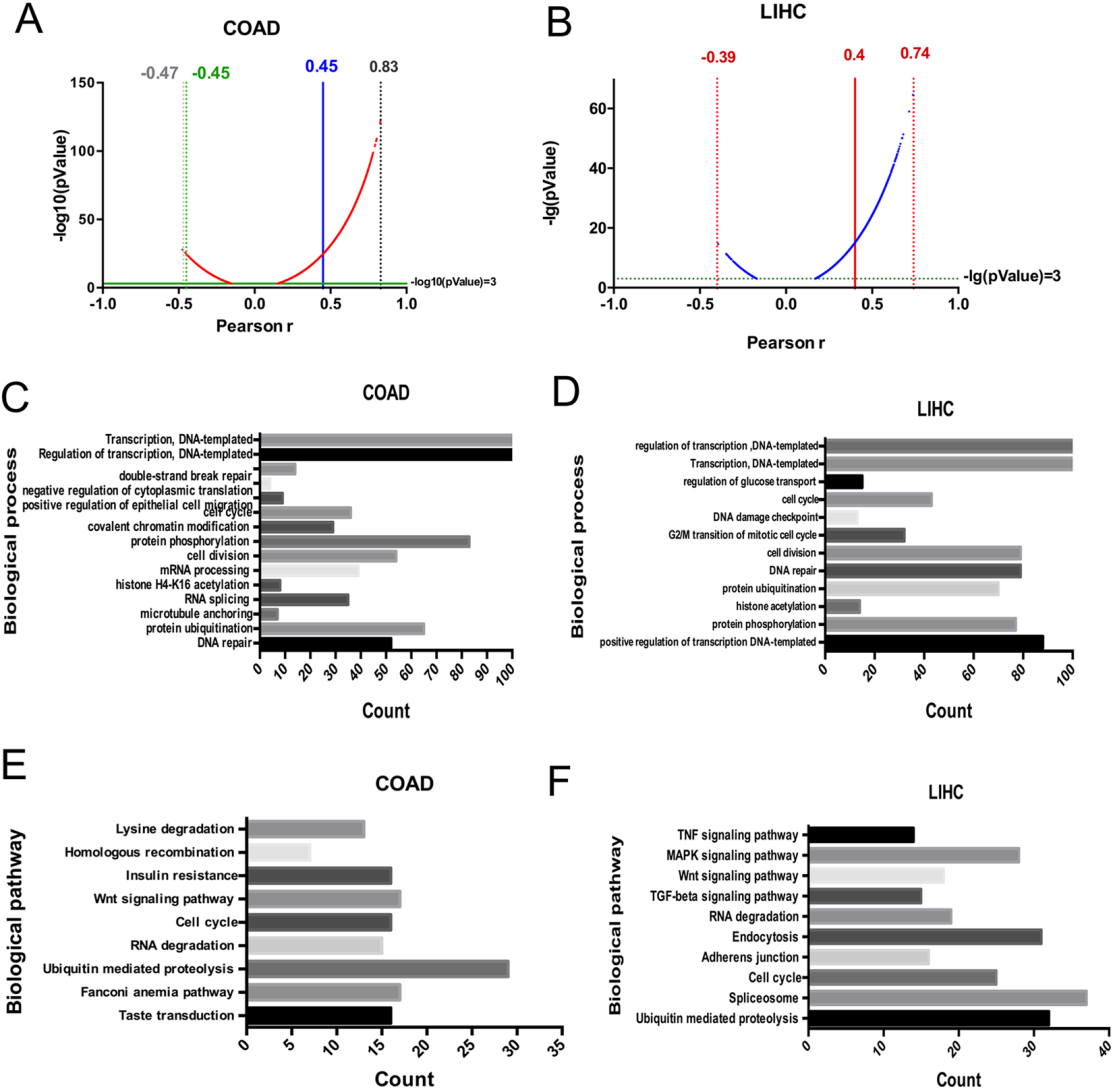
GPNCA and its co-expressed genes participate in tumor associated biological processes and pathways. Co-expression with GPNCA in (**A**) colon adenocarcinoma and (**B**) liver hepatocellular adenocarcinoma. X-axis: Pearson correlation coefficient; Y-axis: -lg (p-value), -lg (p-values) < 3 genes were cut off. Gene ontology (GO) enrichment analysis of GPNCA and its co-expressed genes in (**C**) colon adenocarcinoma and (**D**) liver hepatocellular adenocarcinoma. Genes with absolute Pearson correlation coefficients >0.45 in colon adenocarcinoma and the absolute Pearson correlation coefficients >0.4 were used, and analyses were performed by DAVID. GPNCA and its co-expressed genes were used to perform KEGG analysis in (**E**) colon adenocarcinoma and (**F**) liver hepatocellular adenocarcinoma. *P-values* of all enriched biological processes and pathways were < 0.05.

To study the biological processes and pathways that GPNCA may regulate, GPNCA and its co-expressed genes were assessed. Pearson correlation analysis was performed using the RNA-seq data and cut-off values was set as absolute Pearson r values > 0.45 and p values < 0.001 in colon cancer. Cut-off values were set as absolute Pearson r values > 0.4 and p values < 0.001 in liver cancer (Fig. 6A,B). The screened genes were used to perform GO and KEGG enrichment. In both colon and liver cancer samples, biological processes related to gene transcription and post-transcriptional regulation showed high enrichment, including the regulation of transcription, protein phosphorylation and protein ubiquitination. Biological processes linked to cell growth were also enriched, including cell division, cell cycle and DNA repair (Fig. 6C,D, *P* < *0.05*). KEGG enrichment confirmed the involvement of GPNCA in gene transcription and post-transcriptional modifications, including RNA degradation and ubiquitin-mediated proteolysis. The enrichment in cell cycle and Wnt signaling pathways also corroborated that GPNCA may regulate cell growth (Fig. 6E,F, *P* < *0.05*).

### Down-regulation of GPNCA inhibits cell growth via inhibiting its nearby gene GSK3B

**Figure 7:**
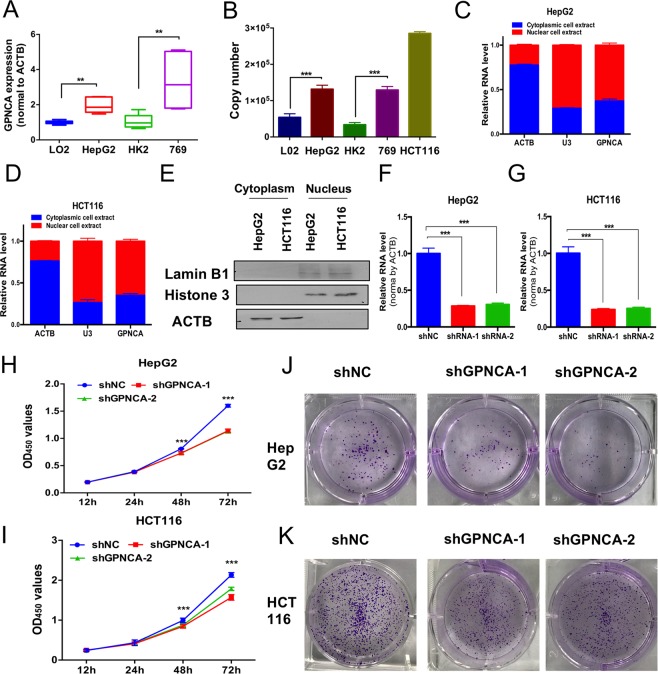
Down-regulation of lncRNA GPNCA inhibits cell growth. (**A**) GPNCA expression assessed by relative qRT-PCR in normal liver cell lines L02, liver cancer cell lines HepG2, normal renal cell lines HK2 and renal cancer lines 769, normalized by house-keeping gene ACTB(n = 6). (**B**) Absolute copy number of GPNCA in L02, HepG2, HK2, 769 and HCT116 determined by absolute qPCR (n = 3), 20 ng total RNA was used for qPCR. (**C**,**D**) GPNCA distributions were analyzed by qRT-PCR through cell cytoplasmic and nuclear RNA purification using HepG2 and HCT116 (n = 3). (**E**) Nuclear/Cytosol fractionation of HCT116 and HepG2 and detected by western blot, Lamin B1 and Histone 3 as nuclear control while ACTB as cytosol control. (**F**,**G**) Stable knockdown efficiency was measured by qRT-PCR in HepG2 and HCT116 cells (n = 3). (**H**,**I**) CCK-8 assays were used to assess the proliferation activity of HepG2 cells and HCT116 cells with or without stable GPNCA knockdown (n = 8). (**J**,**K**) Colony formation assays were performed to analyze the impact of GPNCA on cell proliferation in HepG2 and HCT116 cells. *p < 0.05, **p < 0.01, ***p < *0.001*.

**Figure 8:**
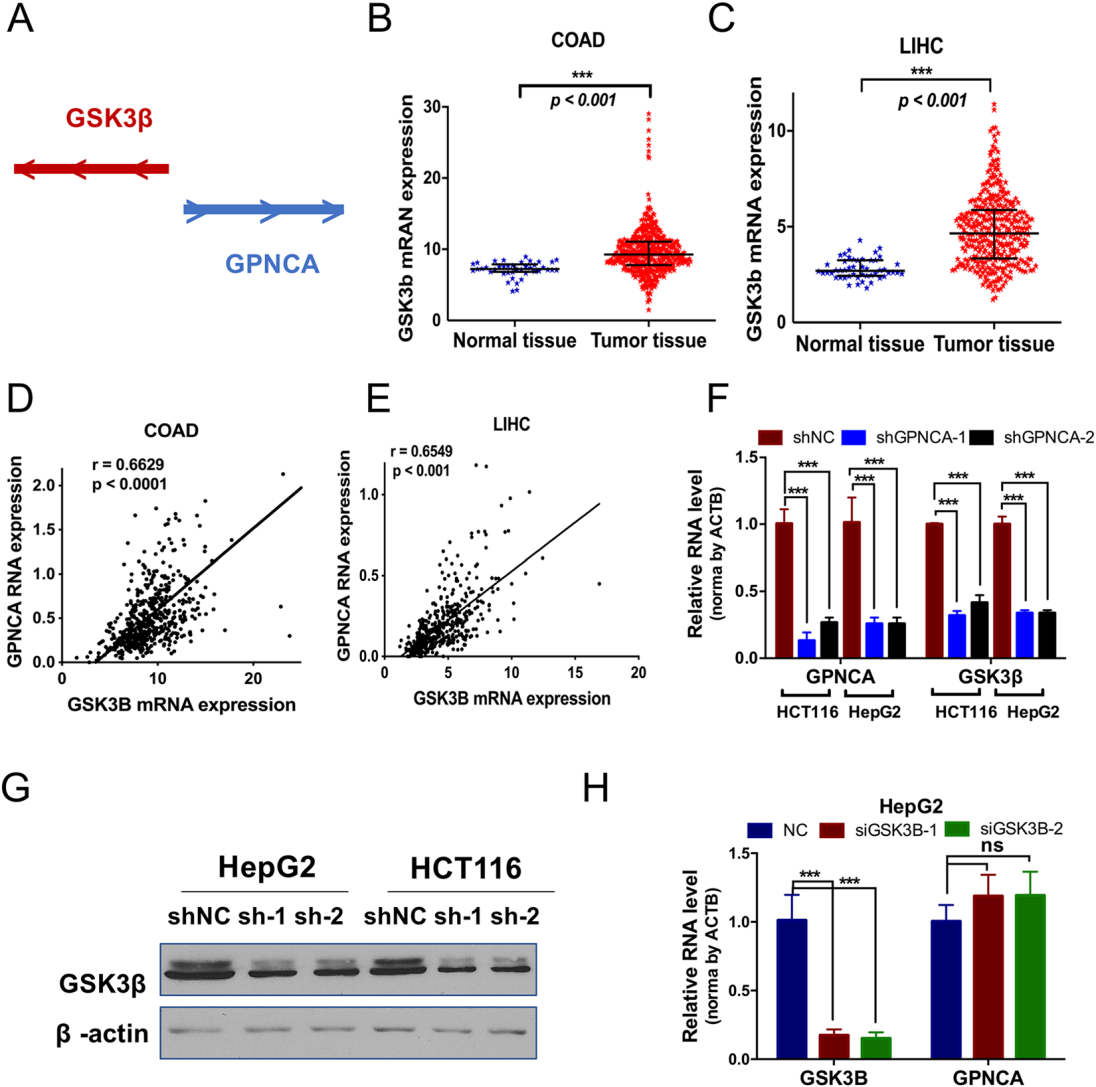
GPNCA regulates GSK3B expression. (**A**) Schema diagram of the relative position and transcript direction of GPNCA and GSK3B. (**B**) GSK3B mRNA expression in COAD: normal tissue (n = 41) and tumor tissue (n = 480). (**C**) GSK3B mRNA expression in LIHC: normal tissue (n = 50) and tumor tissue (n = 374). (**D**,**E**) Gene correlation analysis between GPNCA and GSK3B, using the FPKM values of COAD and LIHC data from TCGA. (**F**) GPNCA and GSK3B expression detected by qPCR in HCT116 and HepG2 cells (n = 3). (**G**) Western blot measured GSK3B protein levels in HepG2 and HCT116 cells with or without GPNCA stable knockdown. (**H**) GPNCA expression was assayed by qPCR in HepG2 cells with or without GSK3B transient knockdown for 48 h (n = 3). *p < 0.05, **p < 0.01, ***p < *0.001*.

We examined the expression of lncRNA GPNCA using the normal human liver cell L02, human liver cancer cell HepG2, human normal renal cell HK2 and human renal cancer cell 769 by SYBR-green relative quantity RT-PCR, which was often used for gene expression detection amongst different cells or tissues. qRT-PCR revealed that GPNCA was overexpressed in the cancer cells (Fig. 7A). In addition, we also did absolute qPCR to detect the copy number of GPNCA and the results also revealed that GPNCA was upregulated in cancer cells (Fig. 7B and Supplemental Fig. S3A). We also performed cell cytoplasm and nuclear RNA purification experiments and showed that GPNCA was mainly distributed in the nucleus, but was also present in the cytoplasm of HepG2 and HCT116 cells (Fig. 7C–E and Supplemental Fig. S3B). These data further support the regulation of gene expression at both the transcriptional and post-transcriptional level by GPNCA. To investigate the role of GPNCA during cell proliferation, HepG2 and HCT116 GPNCA knockdown cells were established using lentivirus shRNA infections. The knockdown efficiency of lncRNA GPNCA was measured by qRT-PCR and showed high efficiency in HepG2 and HCT116 cells (Fig. 7F,G). To elucidate the role of lncRNA GPNCA during tumor cell proliferation, CCK-8 (cell counting kit-8) cell viability assays were performed. The results showed that GPNCA silencing inhibited cell proliferation both in HepG2 and HCT116 cells (Fig. 7H,I). These findings were confirmed in colony formation assays (Fig. 7J,K). Taken together, these data demonstrate that GPNCA silencing inhibits tumor cell growth. As a regulator, it has been reported that the function of divergent lncRNAs which are transcribed in the opposite direction to their nearby protein-coding genes can be inferred from the role of the neighboring adjacent genes^17^. Here, we found GPNCA was a typical divergent lncRNA to GSK3B, whose overexpression promoted tumor growth in many cancers^18,19^ (Fig. 8A). RNA-seq data of COAD and LIHC from TCGA showed that GSK3B were also upregulated in tumor tissue (Fig. 8B,C). In addition, the Pearson’s correlation coefficient of GSK3B and GPNCA was 0.6629 *(p* < *0.001*) in COAD while 0.6549 *(p* < *0.001*) in LIHC (Fig. 8D,E), showing great correlation. What’s more, among 240 COAD patients with high GPNCA expression, 176 patients showed high GSK3B expression. Whilst, 176 amongst 240 COAD patients with low GPNCA expression also showed low GSK3B expression (Supplemental Fig. S4A). Among 187 GPNCA high expression LIHC patients, 137 patients showed high expression of GSK3B while among the 187 GPNCA low expression patients, 137 patients exhibited GSK3B low expression (Supplemental Fig. S4B). These data demonstrated that GPNCA and GSK3B showed consistent expression characteristics in most of the patients. Next, we also performed GO and KEGG analyses and compared the similarities and differences between GPNCA and GSK3B, whose results revealed that most of the Biological processes and biological pathways showed the same, including cell cycle, cell division and DNA repair. However, GPNCA and GSK3B also showed participation in different Biological processes and biological pathways (Supplemental Fig. S4C–F and Supplemental Tables 1–4. These data all indicated that GPNCA positively regulated GSK3B. To make sure, qPCR assays were conducted to detect GSK3B RNA levels in GPNCA stable knockdown HCT116 and HepG2 cells, whose results showed that the mRNA levels of GSK3B were markedly decreased in GPNCA stable knockdown cells (Fig. 8F). Besides, western blot assays were also carried out and the results showed that GSK3B protein were reduced both in HCT116 and HepG2 cells with GPNCA stable knockdown (Fig. 8G and Supplemental Fig. S5A). However, the expressions of GPNCA were not influenced by GSK3B knockdown both in HCT116 and HepG2 cells (Fig. 8H). These data demonstrated that GPNCA regulated tumor cells proliferation by targeting to its nearby protein-coding gene GSK3B and high GPNCA expression was responsible for the upregulation of GSK3B in many tumors.

## Discussion

LncRNAs were largely unexplored for several years^20–23^ during which time an array of proteins were discovered as cancer biomarkers^24–26^. With developments in genomic sequencing technologies, more extensive molecules have been discovered as therapeutic targets for cancer treatment^27^. In the last decade, numerous lncRNAs have been identified as tumor biomarkers^28,29^. However, due to the large numbers of cellular lncRNAs and the tumor complexity^30,31^, their role in cancer development requires further analysis. In this study, we demonstrated that a new lncRNA GPNCA located in the 3q13.33 region is upregulated in many human cancers, including colon cancer and liver cancer, both of which rank amongst the top ten human malignant tumors^2^, suggesting GPNCA represents a biomarker for cancer diagnosis. We verified the upregulation of GPNCA in HepG2 and 769 cells related to L02 and HK2 cells, respectively. Furthermore, we found that the upregulation of GPNCA was associated with the high enrichment of H3k27ac on the GPNCA promoter region via EP300 and GCN5, implicating GPNCA as an oncogene.

We further assessed the prognostic status of colon, liver, breast and renal clear cell cancer patients by downloading data from the *TCGA*, and found that these four cancers with high levels of GPNCA were associated with poor OS. However, due to the not enough good sensibility, GPNCA appeared not a good independent favorable clinical biomarker of prognostic for these cancers. We further found that patients with high neoplasm disease stages showed higher expressions of GPNCA, suggesting its involvement in cancer progression. In addition, we found that colon cancer patients with lymph node metastases or distant metastasis showed GPNCA overexpression. In addition, liver cancer patients with low grade tumor cell differentiation exhibited high levels of GPNCA expression. For the DFS and RFS of colon and liver cancer patients, the overexpression of GPNCA was associated with poor survival. These results demonstrated that GPNCA plays a key role in the progression of colon and liver cancers.

To further investigate the role of GPNCA played in cancer development, the co-expressed genes of GPNCA were used to perform GO and KEGG enrichment analysis. An array of biological processes associated with gene transcription and post-transcriptional modifications were identified, which are known regulatory functions of lncRNAs^11,15,32–36^. Consistent with this finding, cytoplasmic and nuclear RNA purifications revealed that GPNCA was present in the nucleus and cytoplasm. These results were further supported by GO and KEGG analysis, with revealed an additional association of GPNCA with cell division, cell cycle regulation, cell migration and DNA repair. GPNCA silencing in HepG2 and HCT116 cells led to growth arrest, confirming these associations. LncRNA GPNCA is a typical divergent lncRNA that are transcribed in the opposite direction to its nearby protein-coding gene GSK3B. It has been reported that this class lncRNAs could positively regulated their nearby protein-coding genes by binding to regulatory sites on chromatin, such as Evx1as^17^. Herein, we also found that GPNCA as a divergent lncRNA to GSK3B could positively regulated GSK3B at the transcription level by qPCR and western blot, which showed that same pattern as Evx1as. GSK3B were also found overexpression in COAD and LIHC patients and its expression showed high correlation with GPNCA, suggesting that overexpression of GPNCA was the reason for GSK3B upregulation. However, the detail regulating mechanism between GPNCA and GSK3B are needed further study.

In conclusion, we report a new lncRNA GPNCA, the up-regulation of which was related to cancer incidence and poor OS. *In vitro* experiments revealed that GPNCA silencing markedly inhibits tumor cell proliferation via regulation of GSK3B, suggesting that drugs targeted to GPNCA may hold potential considerable value for anti-cancer therapy.

## Methods

### Public data sets

Genome expression and clinical data of colon cancer, liver cancer, renal clear cell cancer, breast cancer, prostate cancer, kidney renal papillary cell cancer, lung adenocarcinoma, lung squamous cell carcinoma and stomach adenocarcinoma were downloaded from The Cancer Genome Atlas (TCGA; https://cancergenome.nih.gov) database using the data transfer tool GDC client (gdc-client_v1.4.0_OSX_x64) according to the instructions. Data integration were conducted using *function* “*merge*” in R environment.

### Analysis of differential gene expression

Gene expression between colon cancer and normal tissue were performed using RNA-seq counts from the TCGA and analyzed using the *R package “edgeR”*. Cut off values were set as absolute log2 (FC) values > 0.85, *p* < *0.001*.

### Analysis of clinical gene expression and survival

Gene expression analysis were performed using RNA-seq FPKM data of normal and tumor tissues using the *Mann-Whitney U test* (Group = 2) or *one-way ANOVA* (Group > 2). For survival analysis, GPNCA expression was dichotomized into “high” and “low” values using time-dependent ROC analysis. Risk analysis was performed using *R package “survival”*. Overall survival (OS), disease-free survival (DFS) and recurrence-free survival (RFS) were analyzed through *Kaplan-Meier* and *log-rank* tests.

### Biological processes and pathway enrichment analysis

Co-expression of GPNCA was analyzed through Pearson correlation coefficients using the “apply” and “cor” in R environment. Co-expressed genes were selected with absolute Pearson correlation coefficients >0.45 in colon cancer, whilst absolute Pearson correlation coefficients >0.4 in liver cancer were used for gene ontology (GO) enrichment and Kyoto encyclopedia of genes and genomes (KEGG) pathway analysis. GO and KEGG analyses were performed using the Database for Annotation, Visualization and Integrated Discovery (DAVID) v6.8^37^.

### Cell culture and treatments

HepG2, 769, HK2 and HCT116 cells were purchased from the Global Bioresource Center (ATCC). L02 cells were kindly provided by the Stem Cell Bank, Chinese Academy of Sciences. HepG2 and L02 cells were maintained in complete DMEM (Gibco, 11965092), HCT116 and 769 cells were cultured in RPMI 1640 (Gibco, A1049101) and HK2 cells were maintained in K-SFM (Gibco, 17005-042). All culture media contained 10% FBS (Biowest, S1810). Cells were incubated in humidified atmosphere with 5% CO2 at 37 °C. To establish the stable knockdown of GPNCA, HepG2 and HCT116 cells were infected with shlncRNA (Target 1: 5′-CCAGAAAGCACATGTTAAAGG-3′; Target 2: 5′-CCTATATTTGATCAGATTACC-3′) lentiviruses. shNC (5′-TTCTCCGAACGTGTCACGTTTC-3′) were used as a negative control (GenePharma, Shanghai). Cells were screened using 2 ug/mL puromycin (Gibco, A11138-03). GSK3B was knockdown by GSK3B special RNAi (Target 1: 5′-CAGCTATACAGACACTAAAGT-3′; Target 2: 5′-GATGAGGTCTATCTTAATC-3′).

### Analysis of *in vitro* gene expression

Gene expression was analyzed by real-time relative quantitative PCR (qRT-PCR). Total RNA was extracted with RNA iso Plus (Takara, D9108B) and RNA concentrations and purity were assessed on a Nanodrop 2000 (Thermo Fisher Scientific, ND-2000). Total RNA (2 ug) was reverse-transcribed using ReverTra Ace qPCR RT kits (TOYOBO, FSQ-101). qRT-PCRs were performed using SYBR® Green Real time PCR Master Mix (TOYOBO, QPK-201). Fold-changes were calculated using the *2*^*−ΔΔCt*^ method and normalized to the housekeeping gene β-actin. Primers used for detecting are as follows: ACTB forward and reverse primers, 5′-TGACGTGGACATCCGCAAAG-3′ and 5′-CTGGAAGGTGGACAGCGAGG-3′; GPNCA forward and reverse primers, 5′-TTACAATGGCTAAGATTTGG-3′ and 5′-ATGTGCTGCTTGTCTTCTG-3′; GSK3B forward and reverse primers, 5′-GGCAGCATGAAAGTTAGCAGA-3′ and 5′-GGCGACCAGTTCTCCTGAATC-3′; U3 forward and reverse primers, 5′-CCACGAGGAAGAGAGGTAGC-3′ and 5′-CACTCAGACCGCGTTCTCTC-3′.

The copy number of GPNCA was measured by absolute quantitative PCR. In brief, a 195 bp characteristic segment of GPNCA was cloned and inserted into the pEASY-Blunt Cloning Vector (Transgen, CB101) and was verified by sequencing. The plasmid was ten-fold diluted as the standard substance and the standard curve was performed by SYBR-green qPCR. Then, the copy number of GPNCA in HepG2, L02, HK2, 769 or HCT116 was verified.

### Analysis of protein level

Protein expression was detected by western blot. Briefly, hepG2 and L02 cells were planted in six well and harvested with RIPA buffer for western blot. Primary antibodies were showed as follows: EP300 (Abcam, ab14984), KAT2A/GCN5 (Abcam, ab208097), H3k27ac (Abcam, ab177178), β-actin (Proteintech, 60008-1-lg), Lamin B1 (CST, #13435) and Histone 3 (Abcam, ab1791).

### Chromatin immunoprecipitation

HepG2 and L02 cells were cultured in 100-mm culture dishes and crosslinked with 1% formaldehyde. Then cells were lysed with cell lysis buffer (50 mM Tris-HCl, 5 mM EDTA, 150 mM NaCl, 1% Triton X-100, 0.1% Deoxycholate and PIC). Next, cells were sonicated and the supernatants were treated with EP300 (Abcam, ab14984), GCN5 (Abcam, ab217876) and IgG (Abcam, ab190475; ab172730) primary antibody for 2 h followed by rotation with 25ul dynabeads protein G for 2 more hours. The DNA fractions were purified and used for next qPCR. Primers are as follows: GPNCA-pro-1 forward and reverse primers, 5′-CGCCTTGCACTTCCCCACT-3′ and 5′-TCCCAGACGCCTGTTACGC-3′; GPNCA-pro-2 forward and reverse primers, 5′-TTTGTTTGCGGCTCCTCCC-3′ and 5′-TTGCCATTGTCGAATGTCTCC-3′.

### Nuclear/cytosol RNA or protein fractionation assay

Cell nuclear and cytosol fractionation was carried out by Nuclear/Cytosol fractionation kit (BioVision, K266-25) according to the manufacturer’s instructions. In short, 200 w HepG2 cells or HCT116 cells were harvested and lysed by 0.2 ml CEBA-A mix buffer for 10 mins. For cytoplasmic RNA extraction, 1 ml RNA iso Plus (Takara, D9108B) was added and centrifuged for 5 min at maximal speed in a microcentrifuge, the supernatant was collected; For cytoplasmic protein extraction, 11 ul of ice-cold Cytosol Extraction Buffer-B was added before centrifugation and then transfer the supernatant fraction to a clean pre-chilled tube. The pellet was then washed with ice-cold PBS containing DTT and Protease Inhibitor cocktail three times. For RNA extraction, the pellet was lysed by 1 ml RNA iso Plus; For protein extraction, 40ul ice-cold Nuclear Extraction Buffer Mix was added and vortex on the highest setting for 15 s and then return the sample to ice for 10 mins, repeat this step for 4 times. Then, samples were centrifuged at full speed for 10 mins and the supernatant was collected.

### Cell counting kit-8 assay

Cell proliferation assays were performed through CCK-8 assays (MCE, K0301) according to the manufacturer’s instructions. Briefly, ~1000 cells were seeded into 96 well cell culture dishes and after 12 h, 24 h, 48 h or 72 h, culture medium was replaced with 110 µl of fresh medium supplemented with 10 µl CCK-8 solution and incubated for 2 h. Measurements were performed on a microplate reader at 450 nm.

### Colony formation assays

For colony formation assays, ~500 HepG2 or ~2000 HCT116 cells with or without stable GPNCA knockdown were seeded into 6-well cell culture plates in the absence of puromycin added and the culture medium was replenished every 2 days. When visible colonies formed, cells were fixed with 1% formaldehyde for 15 min and stained with 0.5% crystal violet for 5 min.

### Statistical analyses

Results are expressed as the mean ± SD or median ± interquartile. Statistical analysis was performed using a *Student’s t-test, Mann-Whitney test, one-way ANOVA* or *log-rank test*. Differences were considered significant for *p-values* < 0.05. Diagrams were plotted using Prism Graph Pad 6.0.

## Supplementary information

Supplementary Information.

## References

[1] Collaborators GS (2018). Measuring progress from 1990 to 2017 and projecting attainment to 2030 of the health-related Sustainable Development Goals for 195 countries and territories: a systematic analysis for the Global Burden of Disease Study 2017. Lancet.

[2] Bray F (2018). Global cancer statistics 2018: GLOBOCAN estimates of incidence and mortality worldwide for 36 cancers in 185 countries. CA Cancer J. Clin..

[3] Hanahan D, Weinberg RA (2011). Hallmarks of cancer: the next generation. Cell.

[4] Potti A (2006). Genomic signatures to guide the use of chemotherapeutics. Nat. Med..

[5] Where is the Future of Drug Discovery for Cancer? *Cell* **168**, 564–565, 10.1016/j.cell.2017.01.032 (2017). 10.1016/j.cell.2017.01.032 28187277

[6] Niu DK, Jiang L (2013). Can ENCODE tell us how much junk DNA we carry in our genome?. Biochem. Biophys. Res. Commun..

[7] Carninci P (2005). The transcriptional landscape of the mammalian genome. Sci..

[8] Ponting CP, Oliver PL, Reik W (2009). Evolution and functions of long noncoding RNAs. Cell.

[9] Liao M (2019). LncRNA EPB41L4A-AS1 regulates glycolysis and glutaminolysis by mediating nucleolar translocation of HDAC2. EBioMedicine.

[10] Huang JZ (2017). A Peptide Encoded by a Putative lncRNA HOXB-AS3 Suppresses Colon Cancer Growth. Mol. Cell.

[11] Wang KC, Chang HY (2011). Molecular mechanisms of long noncoding RNAs. Mol. Cell.

[12] Wilusz JE, Sunwoo H, Spector DL (2009). Long noncoding RNAs: functional surprises from the RNA world. Genes. Dev..

[13] Gupta RA (2010). Long non-coding RNA HOTAIR reprograms chromatin state to promote cancer metastasis. Nat..

[14] Li T (2016). Upregulation of long noncoding RNA ZEB1-AS1 promotes tumor metastasis and predicts poor prognosis in hepatocellular carcinoma. Oncogene.

[15] Wang J (2010). CREB up-regulates long non-coding RNA, HULC expression through interaction with microRNA-372 in liver cancer. Nucleic Acids Res..

[16] Fang C (2017). Long non-coding RNA HNF1A-AS1 mediated repression of miR-34a/SIRT1/p53 feedback loop promotes the metastatic progression of colon cancer by functioning as a competing endogenous RNA. Cancer Lett..

[17] Luo S (2016). Divergent lncRNAs Regulate Gene Expression and Lineage Differentiation in Pluripotent Cells. Cell Stem Cell.

[18] Ghosh JC, Altieri DC (2005). Activation of p53-dependent apoptosis by acute ablation of glycogen synthase kinase-3beta in colorectal cancer cells. Clin. Cancer Res..

[19] Mai W (2009). Deregulated GSK3{beta} sustains gastrointestinal cancer cells survival by modulating human telomerase reverse transcriptase and telomerase. Clin. Cancer Res..

[20] Ohno S (1972). So much “junk” DNA in our genome. Brookhaven Symp. Biol..

[21] Castillo-Davis CI (2005). The evolution of noncoding DNA: how much junk, how much func?. Trends Genet..

[22] Palazzo AF, Lee ES, Non-coding RNA (2015). what is functional and what is junk?. Front. Genet..

[23] Pennisi EG (2012). ENCODE project writes eulogy for junk DNA. Sci..

[24] Rifai N, Gillette MA, Carr SA (2006). Protein biomarker discovery and validation: the long and uncertain path to clinical utility. Nat. Biotechnol..

[25] Matsumoto K, Umitsu M, De Silva DM, Roy A, Bottaro DP (2017). Hepatocyte growth factor/MET in cancer progression and biomarker discovery. Cancer Sci..

[26] Krishnan H (2018). Podoplanin: An emerging cancer biomarker and therapeutic target. Cancer Sci..

[27] Sawyers CL (2008). The cancer biomarker problem. Nat..

[28] Gu J (2018). Downregulation of lncRNA GAS5 confers tamoxifen resistance by activating miR-222 in breast cancer. Cancer Lett..

[29] Wang Q (2016). A novel cell cycle-associated lncRNA, HOXA11-AS, is transcribed from the 5-prime end of the HOXA transcript and is a biomarker of progression in glioma. Cancer Lett..

[30] Hanahan D, Weinberg RA (2000). The hallmarks of cancer. Cell.

[31] Cell editorial, t. Cancer: The Road Ahead. *Cell* **168**, 545–546, 10.1016/j.cell.2017.01.036 (2017). 10.1016/j.cell.2017.01.036 28187271

[32] Dawson MA, Kouzarides T (2012). Cancer epigenetics: from mechanism to therapy. Cell.

[33] Bernstein E, Allis CD (2005). RNA meets chromatin. Genes. Dev..

[34] Gong C, Maquat L (2011). E. lncRNAs transactivate STAU1-mediated mRNA decay by duplexing with 3′ UTRs via Alu elements. Nat..

[35] Wang P (2014). The STAT3-binding long noncoding RNA lnc-DC controls human dendritic cell differentiation. Sci..

[36] Yang F, Zhang H, Mei Y, Wu M (2014). Reciprocal regulation of HIF-1alpha and lincRNA-p21 modulates the Warburg effect. Mol. Cell.

[37] Huang da W, Sherman BT, Lempicki RA (2009). Systematic and integrative analysis of large gene lists using DAVID bioinformatics resources. Nat. Protoc..

