# Supplementary material for: Upregulation of GPNCA is associated with poor prognosis through enhancement of tumor growth via regulating GSK3B
Source: Sci Rep. 2020 Feb 6;10:2044. doi: 10.1038/s41598-020-58729-6 (PMC7004998; doi:10.1038/s41598-020-58729-6)
Supplement: Supplementary file 1 — Supplementary Information. [file 41598_2020_58729_MOESM1_ESM.pdf]

Supplementary Materials for

Upregulation of GPNCA is associated with poor prognosis through enhancement of tumor growth via regulating GSK3B

Weijie Liao[1,2,3&], Fuhai Liu[2,4&], Haowei Zhang [1,2], Weifang Liao[5], Naihan Xu[2,3,4], Weidong Xie[2,3,4], Yaou Zhang[2,3,4, *]

[1]School of Life Sciences, Tsinghua University, Beijing 100084, P.R. China.

[2]Key Lab in Healthy Science and Technology, Division of Life Science, Graduate School at Shenzhen, Tsinghua University, Shenzhen 518055, P.R. China.

[3]State Key Laboratory of Chemical Oncogenomic, Graduate School at Shenzhen, Tsinghua University, Shenzhen, P.R. China.

[4]Open FIESTA Center, Tsinghua University, Shenzhen 518055, P.R. China.

[5]School of biology and pharmaceutical engineering, Wuhan Polytechnic University, Wuhan, 430023, P.R. China.

[&] These authors contributed equally to this work

*Corresponding author: Yaou Zhang, Building L 410C, Key Lab in Healthy Science and Technology, Division of Life Science, Graduate School at Shenzhen, Tsinghua University, Shenzhen, P.R. China. E-mail: zhangyo@sz.tsinghua.edu.cn. Fax: (86) 755-26036884.

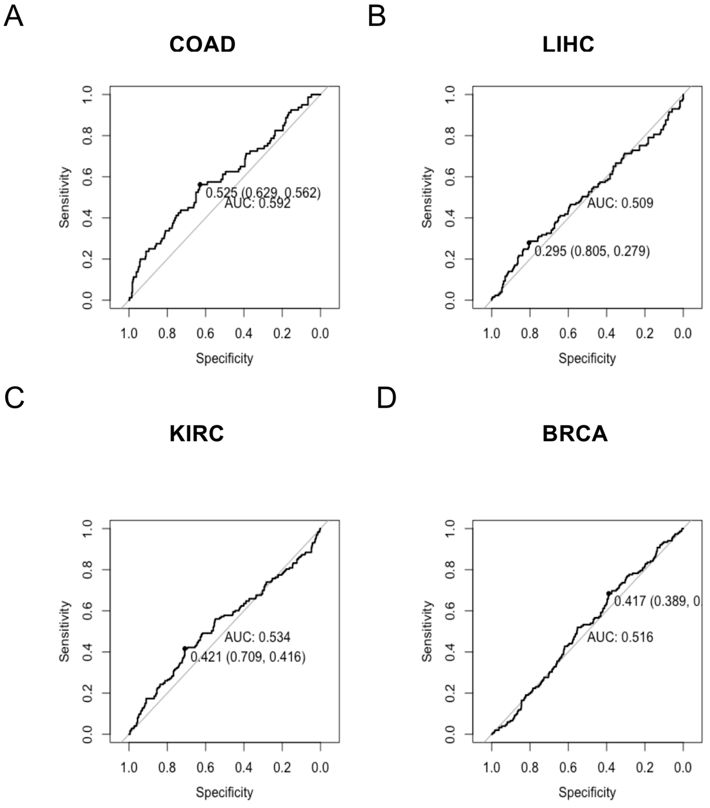

Supplemental Figure S1. High expression of lncRNA GPNCA is associated with poor survival. ROC curve was performed using data of survival status and GPNCA RNA-seq FPKM values of the (A)COAD patients (n = 480), (B) LIHC patients (n = 370), (C)KIRC patients (n = 530) and (D) BRCA patients (n = 1102) by *R package "pROC"*.

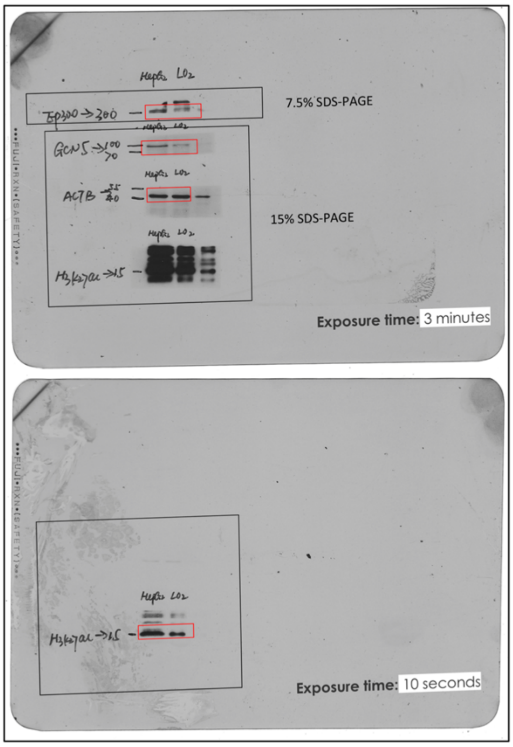

Supplemental Figure S2. Original exposure picture of SDS-PAGE gel for proteins related to figure 5H. 80ug HepG2 and L02 cell lysis were used for western blotting. H3k27ac, ACTB, GCN5 and EP300 proteins were identified. Proteins were separated on 7.5% and 15% SDS-PAGE as marked on above. The lower picture showed the short exposure (10 seconds) and the upper picture showed the long exposure (3mins). The red squares represented the cropped area related to figure 5H.

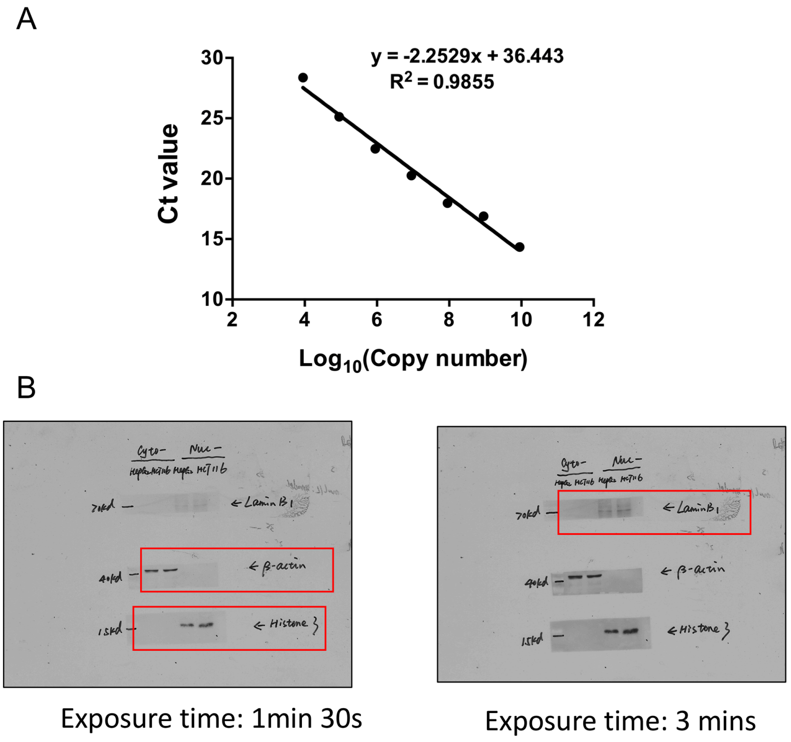

Supplemental Figure S3. Down-regulation of GPNCA inhibits cell growth. (A)The standard curve of GPNCA copy number and Ct value determined by absolute qPCR related to figure 7B**. (B)**Original exposure picture of SDS-PAGE gel for proteins related to figure 7E. 200w HepG2 and HCT116 cells were used for nuclear/cytosol fractionation, Lamin B1 and Histone 3 proteins were identified as nuclear protein marker while ACTB was identified as cytosol protein marker. Proteins were separated on 15% SDS-PAGE. The red squares represented the cropped area related to figure 7E.

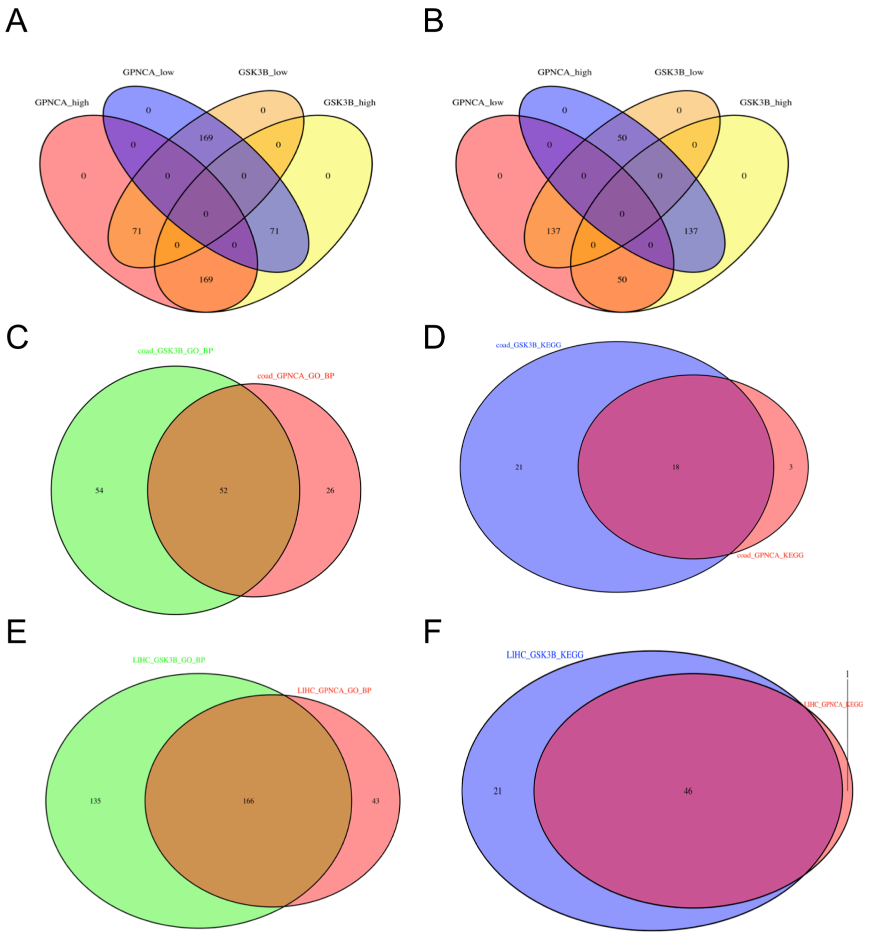

Supplemental Figure S4. GPNCA regulates GSK3B.(A)on one hand,480 COAD patients from TCGA were divided into two parts, the cut-off value was the median of GPNCA RNA-seq FPKM value, on the other hand, 480 COAD patients from TCGA were divided into two parts, the cut-off value was the median of GSK3B RNA-seq FPKM value, then Venn diagram was plotted by *R package "VennDigram"*. (B) 374 LIHC patients from TCGA were divided into two parts according to the median of GPNCA RNA-seq FPKM value, on the other hand, 374 LIHC patients from TCGA were divided into two parts, the cut-off value was the median of GSK3B RNA-seq FPKM value, then Venn diagram was plotted by *R package "VennDigram"*. (C-F) GSK3B and its co-expressed genes were used for GO and KEGG enrichments analyses, Pearson correlation analysis was performed using the RNA-seq data and cut-off values was set as absolute Pearson r values > 0.58 and p values <0.05 in COAD while Pearson r values > 0.42 and p values <0.05 in LIHC, Then Venn diagrams were plotted by *R package "VennDigram"*to compare the GO/KEGG analyses with GPNCA's related to Figure 6C-F. (C) the Biological processes between GPNCA and GSK3B in COAD; (D) The KEGG pathways between GPNCA and GSK3B in COAD; (E) the Biological processes between

GPNCA and GSK3B in LIHC; (D) The KEGG pathways between GPNCA and GSK3B in LIHC.

A

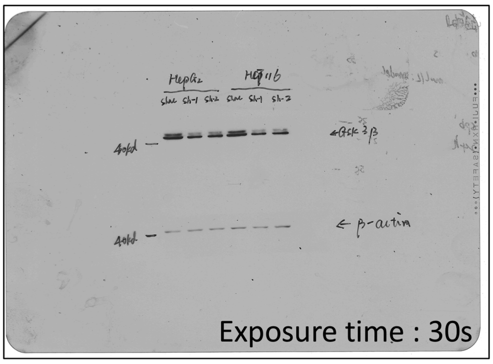

B

>GPNCA

```
GCTGGTGAAACTGCACGGCTGGGCGCCGCCGCCGCTTTGTGATTGGCTGGAAAACTCCGCT
GGGAGCGTACCTCCTTCGGTGATTGGTGAAGAGAAGGGGGCTTCCGAGGACAGTCTGGCCA
GTCAGGACTTCCCCTCCCTCTCCAAGAGTCATCTCTTTCTGTTAAGATGCTGTCTCCAGAAAG
CACATGTTAAAGGACCTATATTTGATCAGATTACCTGGCTCTTGCTTTGAAACACTGATGAAGG
ACTTGGGACAGAACAAAGTGAGTGGAGGACACTTCAATCTAAAGTGATCTTAAAGGAGGAGTA
GTAGGATTTGAAGATGAAGAAAGAAGAGGGGAGGTTAAAAAATGGAGGCAACAGCTGGGTAC
AGTGGCTCACTCCTGTAATTCCAGCACTTTAGGAGGCCAAGGCTGGAGTGCAATGGTGCGAT
CTCGACTCACTGCAACCTCTGCCTCCCAGGTTCAAGCAATTCTCCTGCCTCAGCTTCCCGAG
TAGCTTGGACTACAGAACTGCTTACAATGGCTAAGATTTGGAAGCAACCTAAGTGTCCAACAG
AGGAATGGATAAAGAAAATGTGGTACATATACACAATGGAGTACTATTCCGCCATAAAAAAGA
AAGAGATCCAGTCATTTGCAACAGCATGAATGGAACTGGAGGTCATTAAGTTAAGTAAAATAA
GCCAGGCACAGAAGACAAGCAGCACATTTTCTCACTTACTTGTAGGACATAAAAGTCAAAATG
ATTGGGCTCATGGATATAGAAAGTGGAAGGATGGTTACCAGAGGCTGGGGGGGGGAGTCGG
GGGGAGGTAGAGATGGATAATGGGTACAAAAAATAGTTAGAATGAATAAGACCTATTGATAGC
ACAATAGGGTGACTATTTAAATAATTATATATTTTAAAATAAAGTGTGTAATTGAGTTGTTTGTA
ACTCAAAGGATAAATGCTTGAGGGGATGAAAAAAAAAAAAAAAA
```

Supplemental Figure S5. (A) Original exposure picture of SDS-PAGE gel for proteins related to figure 8G. 60ug total protein of HepG2 and HCT116 cells with or without GPNCA stable knockdown were used for western blot. Proteins were separated on 15% SDS-PAGE. (B) GPNCA transcript sequences.

Supplemental table S1 BPs of GPNCA and GSK3B in COAD related to Supplemental Figure S4C

COAD_GPNCA_BP	COAD_GSK3B_BP
GO: 0006355~regulation of transcription, DNA-templated	GO: 0006351~transcription, DNA-templated

GO: 0006351~transcription, DNA-templated	GO: 0006355~regulation of transcription, DNA-templated
GO: 0006974~cellular response to DNA damage stimulus	GO: 0006468~protein phosphorylation
GO: 0060271~cilium morphogenesis	GO: 0006974~cellular response to DNA damage stimulus
GO: 0042384~cilium assembly	GO: 0016567~protein ubiquitination
GO: 0006281~DNA repair	GO: 0042384~cilium assembly
GO: 0001580~detection of chemical stimulus involved in sensory perception of bitter taste	GO: 0006397~mRNA processing
GO: 0006468~protein phosphorylation	GO: 0060271~cilium morphogenesis
GO: 0016569~covalent chromatin modification	GO: 0042787~protein ubiquitination involved in ubiquitin-dependent protein catabolic process
GO: 0006397~mRNA processing	GO: 0071108~protein K48-linked deubiquitination
GO: 0035194~posttranscriptional gene silencing by RNA	GO: 0000122~negative regulation of transcription from RNA polymerase II promoter
GO: 0016567~protein ubiquitination	GO: 0045893~positive regulation of transcription, DNA-templated
GO: 0071044~histone mRNA catabolic process	GO: 0008380~RNA splicing
GO: 0008380~RNA splicing	GO: 0071044~histone mRNA catabolic process

GO: 0006623~protein targeting to vacuole	GO: 0022604~regulation of cell morphogenesis
GO: 0035278~miRNA mediated inhibition of translation	GO: 0016569~covalent chromatin modification
GO: 0000724~double-strand break repair via homologous recombination	GO: 0006511~ubiquitin-dependent protein catabolic process
GO: 0000732~strand displacement	GO: 0000209~protein polyubiquitination
GO: 0006376~mRNA splice site selection	GO: 0001580~detection of chemical stimulus involved in sensory perception of bitter taste
GO: 0034453~microtubule anchoring	GO: 0035194~posttranscriptional gene silencing by RNA
GO: 0010606~positive regulation of cytoplasmic mRNA processing body assembly	GO: 0036092~phosphatidylinositol-3-phosphate biosynthetic process
GO: 0035058~nonmotile primary cilium assembly	GO: 0035278~miRNA mediated inhibition of translation
GO: 0061512~protein localization to cilium	GO: 0017148~negative regulation of translation
GO: 0035721~intraciliary retrograde transport	GO: 0006357~regulation of transcription from RNA polymerase II promoter
GO: 0042787~protein ubiquitination involved in ubiquitin-dependent protein catabolic process	GO: 0018105~peptidyl-serine phosphorylation
GO: 0050909~sensory perception of taste	GO: 0016926~protein desumoylation
GO: 0060285~cilium-dependent cell motility	GO: 0034453~microtubule anchoring
GO: 0043984~histone H4-K16 acetylation	GO: 0007032~endosome organization

GO: 0006661~phosphatidylinositol biosynthetic process	GO: 0032886~regulation of microtubule-based process
GO: 0017148~negative regulation of translation	GO: 0043984~histone H4-K16 acetylation
GO: 0030521~androgen receptor signaling pathway	GO: 0016055~Wnt signaling pathway
GO: 0007099~centriole replication	GO: 0006886~intracellular protein transport
GO: 0006511~ubiquitin-dependent protein catabolic process	GO: 0007049~cell cycle
GO: 0016926~protein desumoylation	GO: 0051301~cell division
GO: 0036092~phosphatidylinositol-3-phosphate biosynthetic process	GO: 0045892~negative regulation of transcription, DNA-templated
GO: 0000381~regulation of alternative mRNA splicing, via spliceosome	GO: 0010608~posttranscriptional regulation of gene expression
GO: 1903887~motile primary cilium assembly	GO: 0050909~sensory perception of taste
GO: 0032886~regulation of microtubule-based process	GO: 0043484~regulation of RNA splicing
GO: 0006310~DNA recombination	GO: 0070536~protein K63-linked deubiquitination
GO: 0000209~protein polyubiquitination	GO: 0010606~positive regulation of cytoplasmic mRNA processing body assembly
GO: 0043484~regulation of RNA splicing	GO: 0017196~N-terminal peptidyl-methionine acetylation
GO: 0000731~DNA synthesis involved in DNA repair	GO: 2000114~regulation of establishment of cell polarity

GO: 0071108~protein K48-linked deubiquitination	GO: 0072659~protein localization to plasma membrane
GO: 0006396~RNA processing	GO: 0070423~nucleotide-binding oligomerization domain containing signaling pathway
GO: 0016925~protein sumoylation	GO: 0007173~epidermal growth factor receptor signaling pathway
GO: 0009411~response to UV	GO: 0009791~post-embryonic development
GO: 0022604~regulation of cell morphogenesis	GO: 0006281~DNA repair
GO: 0045893~positive regulation of transcription, DNA-templated	GO: 0043087~regulation of GTPase activity
GO: 0006260~DNA replication	GO: 0007264~small GTPase mediated signal transduction
GO: 2000651~positive regulation of sodium ion transmembrane transporter activity	GO: 0007030~Golgi organization
GO: 0018344~protein geranylgeranylation	GO: 0035280~miRNA loading onto RISC involved in gene silencing by miRNA
GO: 0010212~response to ionizing radiation	GO: 2000766~negative regulation of cytoplasmic translation
GO: 0035023~regulation of Rho protein signal transduction	GO: 0019054~modulation by virus of host process
GO: 0090305~nucleic acid phosphodiester bond hydrolysis	GO: 0031123~RNA 3'-end processing
GO: 0023014~signal transduction by protein phosphorylation	GO: 0030521~androgen receptor signaling pathway

GO: 0060213~positive regulation of nuclear-transcribed mRNA poly(A) tail shortening	GO: 0015031~protein transport
GO: 0090161~Golgi ribbon formation	GO: 0006661~phosphatidylinositol biosynthetic process
GO: 0006349~regulation of gene expression by genetic imprinting	GO: 0048268~clathrin coat assembly
GO: 0043981~histone H4-K5 acetylation	GO: 0031054~pre-miRNA processing
GO: 0007292~female gamete generation	GO: 0019827~stem cell population maintenance
GO: 0043982~histone H4-K8 acetylation	GO: 0035329~hippo signaling
GO: 0010608~posttranscriptional regulation of gene expression	GO: 0007010~cytoskeleton organization
GO: 0007049~cell cycle	GO: 0006211~5-methylcytosine catabolic process
GO: 0010634~positive regulation of epithelial cell migration	GO: 0071139~resolution of recombination intermediates
GO: 0007223~Wnt signaling pathway, calcium modulating pathway	GO: 0032880~regulation of protein localization
GO: 0051301~cell division	GO: 1900246~positive regulation of RIG-I signaling pathway
GO: 0006357~regulation of transcription from RNA polymerase II promoter	GO: 0060736~prostate gland growth
GO: 0006211~5-methylcytosine catabolic process	GO: 0006623~protein targeting to vacuole
GO: 0071596~ubiquitin-dependent protein catabolic process via the N-end rule pathway	GO: 0035196~production of miRNAs involved in gene silencing by miRNA

GO: 0071139~resolution of recombination intermediates	GO: 0048015~phosphatidylinositol-mediated signaling
GO: 0019054~modulation by virus of host process	GO: 0035556~intracellular signal transduction
GO: 0035280~miRNA loading onto RISC involved in gene silencing by miRNA	GO: 0023014~signal transduction by protein phosphorylation
GO: 2000766~negative regulation of cytoplasmic translation	GO: 0032508~DNA duplex unwinding
GO: 0051893~regulation of focal adhesion assembly	GO: 0035264~multicellular organism growth
GO: 2000114~regulation of establishment of cell polarity	GO: 0000226~microtubule cytoskeleton organization
GO: 0006302~double-strand break repair	GO: 0048008~platelet-derived growth factor receptor signaling pathway
GO: 0006607~NLS-bearing protein import into nucleus	GO: 0016579~protein deubiquitination
GO: 0000278~mitotic cell cycle	GO: 0032465~regulation of cytokinesis
	GO: 0080182~histone H3-K4 trimethylation
	GO: 0045944~positive regulation of transcription from RNA polymerase II promoter
	GO: 0000381~regulation of alternative mRNA splicing, via spliceosome
	GO: 0018107~peptidyl-threonine phosphorylation

	GO: 0061088~regulation of sequestering of zinc ion
	GO: 0070102~interleukin-6-mediated signaling pathway
	GO: 0070198~protein localization to chromosome, telomeric region
	GO: 0048278~vesicle docking
	GO: 0006607~NLS-bearing protein import into nucleus
	GO: 0000724~double-strand break repair via homologous recombination
	GO: 0030100~regulation of endocytosis
	GO: 1990090~cellular response to nerve growth factor stimulus
	GO: 0043982~histone H4-K8 acetylation
	GO: 0043981~histone H4-K5 acetylation
	GO: 0032924~activin receptor signaling pathway
	GO: 0090625~mRNA cleavage involved in gene silencing by siRNA
	GO: 0038028~insulin receptor signaling pathway via phosphatidylinositol 3-kinase
	GO: 0043124~negative regulation of I-kappaB kinase/NF-kappaB signaling
	GO: 0002092~positive regulation of receptor internalization

	GO: 0048705~skeletal system morphogenesis
	GO: 0035871~protein K11-linked deubiquitination
	GO: 0035721~intraciliary retrograde transport
	GO: 2000637~positive regulation of gene silencing by miRNA
	GO: 0051146~striated muscle cell differentiation
	GO: 0051893~regulation of focal adhesion assembly
	GO: 0006893~Golgi to plasma membrane transport
	GO: 0006376~mRNA splice site selection
	GO: 0042147~retrograde transport, endosome to Golgi

Supplemental table S2 KEGG pathways of GPNCA and GSK3B in COAD related to Supplemental Figure S4D

COAD_GPNCA_KEGG	COAD_GSK3B_KEGG
hsa04742: Taste transduction	hsa04120: Ubiquitin mediated proteolysis
hsa03460: Fanconi anemia pathway	hsa04742: Taste transduction
hsa04120: Ubiquitin mediated proteolysis	hsa05223: Non-small cell lung cancer
hsa00310: Lysine degradation	hsa05220: Chronic myeloid leukemia
hsa04070: Phosphatidylinositol signaling system	hsa04550: Signaling pathways regulating pluripotency of stem cells

hsa00562: Inositol phosphate metabolism	hsa04012: ErbB signaling pathway
hsa03018: RNA degradation	hsa04068: FoxO signaling pathway
hsa05223: Non-small cell lung cancer	hsa04910: Insulin signaling pathway
hsa03440: Homologous recombination	hsa05213: Endometrial cancer
hsa04931: Insulin resistance	hsa04931: Insulin resistance
hsa04550: Signaling pathways regulating pluripotency of stem cells	hsa05212: Pancreatic cancer
hsa04130: SNARE interactions in vesicular transport	hsa04914: Progesterone-mediated oocyte maturation
hsa04910: Insulin signaling pathway	hsa04010: MAPK signaling pathway
hsa05213: Endometrial cancer	hsa04917: Prolactin signaling pathway
hsa04917: Prolactin signaling pathway	hsa04722: Neurotrophin signaling pathway
hsa04110: Cell cycle	hsa05210: Colorectal cancer
hsa03450: Non-homologous end-joining	hsa04070: Phosphatidylinositol signaling system
hsa04810: Regulation of actin cytoskeleton	hsa03460: Fanconi anemia pathway
hsa04012: ErbB signaling pathway	hsa05215: Prostate cancer
hsa04310: Wnt signaling pathway	hsa05214: Glioma
hsa04150: mTOR signaling pathway	hsa03018: RNA degradation
	hsa05211: Renal cell carcinoma
	hsa04130: SNARE interactions in vesicular transport
	hsa04144: Endocytosis

	hsa04320: Dorso-ventral axis formation
	hsa00562: Inositol phosphate metabolism
	hsa04390: Hippo signaling pathway
	hsa04810: Regulation of actin cytoskeleton
	hsa05166: HTLV-I infection
	hsa04710: Circadian rhythm
	hsa03015: mRNA surveillance pathway
	hsa05161: Hepatitis B
	hsa04150: mTOR signaling pathway
	hsa04110: Cell cycle
	hsa04310: Wnt signaling pathway
	hsa04350: TGF-beta signaling pathway
	hsa05205: Proteoglycans in cancer
	hsa05231: Choline metabolism in cancer
	hsa00510: N-Glycan biosynthesis

Supplemental table S3 BPs of GPNCA and GSK3B in LIHC related to Supplemental Figure S4E

LIHC_GPNCA_GO_BP	LIHC_GSK3B_GO_BP
GO: 0006351~transcription, DNA-templated	GO: 0006351~transcription, DNA-templated
GO: 0006355~regulation of transcription, DNA-templated	GO: 0006355~regulation of transcription, DNA-templated

GO: 0006281~DNA repair	GO: 0006974~cellular response to DNA damage stimulus
GO: 0000398~mRNA splicing, via spliceosome	GO: 0016925~protein sumoylation
GO: 0006260~DNA replication	GO: 0006281~DNA repair
GO: 0006974~cellular response to DNA damage stimulus	GO: 0016032~viral process
GO: 0006397~mRNA processing	GO: 0016569~covalent chromatin modification
GO: 0008380~RNA splicing	GO: 0000398~mRNA splicing, via spliceosome
GO: 0016925~protein sumoylation	GO: 0051301~cell division
GO: 0051301~cell division	GO: 0006260~DNA replication
GO: 0006406~mRNA export from nucleus	GO: 0006397~mRNA processing
GO: 0016569~covalent chromatin modification	GO: 0006406~mRNA export from nucleus
GO: 0006357~regulation of transcription from RNA polymerase II promoter	GO: 0007062~sister chromatid cohesion
GO: 0042384~cilium assembly	GO: 0042787~protein ubiquitination involved in ubiquitin-dependent protein catabolic process
GO: 0007067~mitotic nuclear division	GO: 0016567~protein ubiquitination
GO: 0051298~centrosome duplication	GO: 0045893~positive regulation of transcription, DNA-templated
GO: 0019827~stem cell population maintenance	GO: 0015031~protein transport

GO: 0006396~RNA processing	GO: 0000724~double-strand break repair via homologous recombination
GO: 0000122~negative regulation of transcription from RNA polymerase II promoter	GO: 0098609~cell-cell adhesion
GO: 0000724~double-strand break repair via homologous recombination	GO: 0000122~negative regulation of transcription from RNA polymerase II promoter
GO: 0016567~protein ubiquitination	GO: 0006357~regulation of transcription from RNA polymerase II promoter
GO: 0060271~cilium morphogenesis	GO: 0006886~intracellular protein transport
GO: 0016032~viral process	GO: 0042384~cilium assembly
GO: 0006303~double-strand break repair via nonhomologous end joining	GO: 0006468~protein phosphorylation
GO: 0043984~histone H4-K16 acetylation	GO: 0008380~RNA splicing
GO: 0006409~tRNA export from nucleus	GO: 0000086~G2/M transition of mitotic cell cycle
GO: 0010827~regulation of glucose transport	GO: 0000278~mitotic cell cycle
GO: 0045893~positive regulation of transcription, DNA-templated	GO: 0006338~chromatin remodeling
GO: 1901796~regulation of signal transduction by p53 class mediator	GO: 1901796~regulation of signal transduction by p53 class mediator
GO: 0000381~regulation of alternative mRNA splicing, via spliceosome	GO: 0042147~retrograde transport, endosome to Golgi

GO: 0000732~strand displacement	GO: 0045944~positive regulation of transcription from RNA polymerase II promoter
GO: 1900034~regulation of cellular response to heat	GO: 0060271~cilium morphogenesis
GO: 0036297~interstrand cross-link repair	GO: 0045892~negative regulation of transcription, DNA-templated
GO: 0000731~DNA synthesis involved in DNA repair	GO: 0000731~DNA synthesis involved in DNA repair
GO: 0031047~gene silencing by RNA	GO: 0007067~mitotic nuclear division
GO: 0006405~RNA export from nucleus	GO: 0006409~tRNA export from nucleus
GO: 0045892~negative regulation of transcription, DNA-templated	GO: 1900034~regulation of cellular response to heat
GO: 0007062~sister chromatid cohesion	GO: 0043044~ATP-dependent chromatin remodeling
GO: 0006468~protein phosphorylation	GO: 0000289~nuclear-transcribed mRNA poly(A) tail shortening
GO: 0010467~gene expression	GO: 0018105~peptidyl-serine phosphorylation
GO: 0016573~histone acetylation	GO: 0006396~RNA processing
GO: 0000086~G2/M transition of mitotic cell cycle	GO: 0006303~double-strand break repair via nonhomologous end joining
GO: 0007077~mitotic nuclear envelope disassembly	GO: 0051298~centrosome duplication
GO: 0000077~DNA damage checkpoint	GO: 0019827~stem cell population maintenance

GO: 0042787~protein ubiquitination involved in ubiquitin-dependent protein catabolic process	GO: 0000732~strand displacement
GO: 0016926~protein desumoylation	GO: 0010827~regulation of glucose transport
GO: 0075733~intracellular transport of virus	GO: 0075733~intracellular transport of virus
GO: 0007049~cell cycle	GO: 0006511~ubiquitin-dependent protein catabolic process
GO: 0006338~chromatin remodeling	GO: 0007077~mitotic nuclear envelope disassembly
GO: 0017148~negative regulation of translation	GO: 0031047~gene silencing by RNA
GO: 0045944~positive regulation of transcription from RNA polymerase II promoter	GO: 0018107~peptidyl-threonine phosphorylation
GO: 0010212~response to ionizing radiation	GO: 0007030~Golgi organization
GO: 0007099~centriole replication	GO: 0007049~cell cycle
GO: 0043981~histone H4-K5 acetylation	GO: 0006888~ER to Golgi vesicle-mediated transport
GO: 0043982~histone H4-K8 acetylation	GO: 0006405~RNA export from nucleus
GO: 0010501~RNA secondary structure unwinding	GO: 0032508~DNA duplex unwinding
GO: 0000375~RNA splicing, via transesterification reactions	GO: 0000910~cytokinesis
GO: 0048025~negative regulation of mRNA splicing, via spliceosome	GO: 0007080~mitotic metaphase plate congression
GO: 0051726~regulation of cell cycle	GO: 0051568~histone H3-K4 methylation

GO: 0018105~peptidyl-serine phosphorylation	GO: 0043984~histone H4-K16 acetylation
GO: 0090305~nucleic acid phosphodiester bond hydrolysis	GO: 0043161~proteasome-mediated ubiquitin-dependent protein catabolic process
GO: 0006511~ubiquitin-dependent protein catabolic process	GO: 0006366~transcription from RNA polymerase II promoter
GO: 0009411~response to UV	GO: 0030521~androgen receptor signaling pathway
GO: 0035058~nonmotile primary cilium assembly	GO: 0016573~histone acetylation
GO: 0031297~replication fork processing	GO: 0006661~phosphatidylinositol biosynthetic process
GO: 0007020~microtubule nucleation	GO: 0048025~negative regulation of mRNA splicing, via spliceosome
GO: 0018107~peptidyl-threonine phosphorylation	GO: 0090503~RNA phosphodiester bond hydrolysis, exonucleolytic
GO: 0032508~DNA duplex unwinding	GO: 0000209~protein polyubiquitination
GO: 0051292~nuclear pore complex assembly	GO: 0006369~termination of RNA polymerase II transcription
GO: 0031124~mRNA 3'-end processing	GO: 0090307~mitotic spindle assembly
GO: 0043484~regulation of RNA splicing	GO: 0010467~gene expression
GO: 0000281~mitotic cytokinesis	GO: 0042795~snRNA transcription from RNA polymerase II promoter
GO: 0051568~histone H3-K4 methylation	GO: 0006302~double-strand break repair
GO: 0000070~mitotic sister chromatid segregation	GO: 0000082~G1/S transition of mitotic cell cycle

GO: 0042795~snRNA transcription from RNA polymerase II promoter	GO: 0006607~NLS-bearing protein import into nucleus
GO: 0006369~termination of RNA polymerase II transcription	GO: 0006470~protein dephosphorylation
GO: 0090307~mitotic spindle assembly	GO: 0031124~mRNA 3'-end processing
GO: 0000723~telomere maintenance	GO: 0000186~activation of MAPKK activity
GO: 0051571~positive regulation of histone H3-K4 methylation	GO: 0006367~transcription initiation from RNA polymerase II promoter
GO: 0031053~primary miRNA processing	GO: 0006893~Golgi to plasma membrane transport
GO: 0051297~centrosome organization	GO: 0007059~chromosome segregation
GO: 0001701~in utero embryonic development	GO: 0051292~nuclear pore complex assembly
GO: 0061512~protein localization to cilium	GO: 0051726~regulation of cell cycle
GO: 0046600~negative regulation of centriole replication	GO: 0006261~DNA-dependent DNA replication
GO: 0021532~neural tube patterning	GO: 0036297~interstrand cross-link repair
GO: 0006310~DNA recombination	GO: 0007163~establishment or maintenance of cell polarity
GO: 0007064~mitotic sister chromatid cohesion	GO: 0000281~mitotic cytokinesis
GO: 0016578~histone deubiquitination	GO: 0000729~DNA double-strand break processing
GO: 0000289~nuclear-transcribed mRNA poly(A) tail shortening	GO: 0034058~endosomal vesicle fusion

GO: 0035721~intraciliary retrograde transport	GO: 0016070~RNA metabolic process
GO: 0008589~regulation of smoothened signaling pathway	GO: 0017148~negative regulation of translation
GO: 0071108~protein K48-linked deubiquitination	GO: 0035518~histone H2A monoubiquitination
GO: 0000278~mitotic cell cycle	GO: 0048024~regulation of mRNA splicing, via spliceosome
GO: 0030521~androgen receptor signaling pathway	GO: 0032465~regulation of cytokinesis
GO: 0006302~double-strand break repair	GO: 0048511~rhythmic process
GO: 0006368~transcription elongation from RNA polymerase II promoter	GO: 0016575~histone deacetylation
GO: 0043968~histone H2A acetylation	GO: 0000077~DNA damage checkpoint
GO: 0080182~histone H3-K4 trimethylation	GO: 0000381~regulation of alternative mRNA splicing, via spliceosome
GO: 0000729~DNA double-strand break processing	GO: 0007010~cytoskeleton organization
GO: 0019985~translesion synthesis	GO: 0043982~histone H4-K8 acetylation
GO: 0006306~DNA methylation	GO: 0043981~histone H4-K5 acetylation
GO: 0051090~regulation of sequence-specific DNA binding transcription factor activity	GO: 0046777~protein autophosphorylation
GO: 0007094~mitotic spindle assembly checkpoint	GO: 0006283~transcription-coupled nucleotide-excision repair
GO: 0035194~posttranscriptional gene silencing by RNA	GO: 0016192~vesicle-mediated transport

GO: 0071479~cellular response to ionizing radiation	GO: 0043087~regulation of GTPase activity
GO: 0043967~histone H4 acetylation	GO: 0051225~spindle assembly
GO: 0007059~chromosome segregation	GO: 0035329~hippo signaling
GO: 0006366~transcription from RNA polymerase II promoter	GO: 0008333~endosome to lysosome transport
GO: 0030518~intracellular steroid hormone receptor signaling pathway	GO: 0072383~plus-end-directed vesicle transport along microtubule
GO: 0006921~cellular component disassembly involved in execution phase of apoptosis	GO: 0033962~cytoplasmic mRNA processing body assembly
GO: 0007093~mitotic cell cycle checkpoint	GO: 0090305~nucleic acid phosphodiester bond hydrolysis
GO: 0040001~establishment of mitotic spindle localization	GO: 0016579~protein deubiquitination
GO: 0051225~spindle assembly	GO: 0007032~endosome organization
GO: 0006370~7-methylguanosine mRNA capping	GO: 0051571~positive regulation of histone H3-K4 methylation
GO: 0006361~transcription initiation from RNA polymerase I promoter	GO: 0006337~nucleosome disassembly
GO: 0048024~regulation of mRNA splicing, via spliceosome	GO: 1904261~positive regulation of basement membrane assembly involved in embryonic body morphogenesis
GO: 0035278~miRNA mediated inhibition of translation	GO: 1902017~regulation of cilium assembly
GO: 0032465~regulation of cytokinesis	GO: 0051865~protein autoubiquitination

GO: 0016579~protein deubiquitination	GO: 0007179~transforming growth factor beta receptor signaling pathway
GO: 0006376~mRNA splice site selection	GO: 0044030~regulation of DNA methylation
GO: 0090503~RNA phosphodiester bond hydrolysis, exonucleolytic	GO: 0000375~RNA splicing, via transesterification reactions
GO: 0000186~activation of MAPKK activity	GO: 0006306~DNA methylation
GO: 0043044~ATP-dependent chromatin remodeling	GO: 0009411~response to UV
GO: 0033962~cytoplasmic mRNA processing body assembly	GO: 0051168~nuclear export
GO: 0051457~maintenance of protein location in nucleus	GO: 0016926~protein desumoylation
GO: 0071539~protein localization to centrosome	GO: 0035196~production of miRNAs involved in gene silencing by miRNA
GO: 0007126~meiotic nuclear division	GO: 0000059~protein import into nucleus, docking
GO: 0006362~transcription elongation from RNA polymerase I promoter	GO: 0006623~protein targeting to vacuole
GO: 0046777~protein autophosphorylation	GO: 0090630~activation of GTPase activity
GO: 0006264~mitochondrial DNA replication	GO: 0006897~endocytosis
GO: 0034058~endosomal vesicle fusion	GO: 0031532~actin cytoskeleton reorganization
GO: 0016070~RNA metabolic process	GO: 0007173~epidermal growth factor receptor signaling pathway

GO: 0035562~negative regulation of chromatin binding	GO: 0048208~COPII vesicle coating
GO: 0032886~regulation of microtubule-based process	GO: 0043966~histone H3 acetylation
GO: 0070816~phosphorylation of RNA polymerase II C-terminal domain	GO: 0007076~mitotic chromosome condensation
GO: 0051304~chromosome separation	GO: 0006301~postreplication repair
GO: 0010032~meiotic chromosome condensation	GO: 0006298~mismatch repair
GO: 0043966~histone H3 acetylation	GO: 0007018~microtubule-based movement
GO: 0000380~alternative mRNA splicing, via spliceosome	GO: 0006368~transcription elongation from RNA polymerase II promoter
GO: 0031573~intra-S DNA damage checkpoint	GO: 0008589~regulation of smoothened signaling pathway
GO: 0001843~neural tube closure	GO: 0071479~cellular response to ionizing radiation
GO: 0007080~mitotic metaphase plate congression	GO: 0036258~multivesicular body assembly
GO: 0022604~regulation of cell morphogenesis	GO: 0023014~signal transduction by protein phosphorylation
GO: 0006363~termination of RNA polymerase I transcription	GO: 0035058~nonmotile primary cilium assembly
GO: 0034644~cellular response to UV	GO: 0006891~intra-Golgi vesicle-mediated transport
GO: 0031572~G2 DNA damage checkpoint	GO: 0010717~regulation of epithelial to mesenchymal transition

GO: 0000245~spliceosomal complex assembly	GO: 0032801~receptor catabolic process
GO: 0006301~postreplication repair	GO: 0006890~retrograde vesicle-mediated transport, Golgi to ER
GO: 0045444~fat cell differentiation	GO: 0010212~response to ionizing radiation
GO: 0016575~histone deacetylation	GO: 0000723~telomere maintenance
GO: 0006283~transcription-coupled nucleotide-excision repair	GO: 0010507~negative regulation of autophagy
GO: 0030953~astral microtubule organization	GO: 0019985~translesion synthesis
GO: 0010606~positive regulation of cytoplasmic mRNA processing body assembly	GO: 0032147~activation of protein kinase activity
GO: 0006999~nuclear pore organization	GO: 0000722~telomere maintenance via recombination
GO: 0000395~mRNA 5'-splice site recognition	GO: 2000786~positive regulation of autophagosome assembly
GO: 0048478~replication fork protection	GO: 0031053~primary miRNA processing
GO: 1901990~regulation of mitotic cell cycle phase transition	GO: 0006353~DNA-templated transcription, termination
GO: 0010608~posttranscriptional regulation of gene expression	GO: 0007099~centriole replication
GO: 0033169~histone H3-K9 demethylation	GO: 0010606~positive regulation of cytoplasmic mRNA processing body assembly
GO: 0051168~nuclear export	GO: 0021532~neural tube patterning
GO: 0006289~nucleotide-excision repair	GO: 0006999~nuclear pore organization

GO: 0006367~transcription initiation from RNA polymerase II promoter	GO: 0006896~Golgi to vacuole transport
GO: 0000209~protein polyubiquitination	GO: 0030953~astral microtubule organization
GO: 0048568~embryonic organ development	GO: 0006378~mRNA polyadenylation
GO: 2000114~regulation of establishment of cell polarity	GO: 0044255~cellular lipid metabolic process
GO: 2000785~regulation of autophagosome assembly	GO: 0006289~nucleotide-excision repair
GO: 0007276~gamete generation	GO: 0051149~positive regulation of muscle cell differentiation
GO: 0036092~phosphatidylinositol-3-phosphate biosynthetic process	GO: 0008360~regulation of cell shape
GO: 0016055~Wnt signaling pathway	GO: 0007265~Ras protein signal transduction
GO: 0000226~microtubule cytoskeleton organization	GO: 0035264~multicellular organism growth
GO: 0010586~miRNA metabolic process	GO: 0060996~dendritic spine development
GO: 0060831~smoothened signaling pathway involved in dorsal/ventral neural tube patterning	GO: 0006622~protein targeting to lysosome
GO: 0051988~regulation of attachment of spindle microtubules to kinetochore	GO: 0045197~establishment or maintenance of epithelial cell apical/basal polarity
GO: 0061014~positive regulation of mRNA catabolic process	GO: 0031054~pre-miRNA processing
GO: 0033147~negative regulation of intracellular estrogen receptor signaling pathway	GO: 0048268~clathrin coat assembly

GO: 0010717~regulation of epithelial to mesenchymal transition	GO: 0051028~mRNA transport
GO: 0031937~positive regulation of chromatin silencing	GO: 0051090~regulation of sequence-specific DNA binding transcription factor activity
GO: 0051684~maintenance of Golgi location	GO: 0022604~regulation of cell morphogenesis
GO: 0071139~resolution of recombination intermediates	GO: 0000226~microtubule cytoskeleton organization
GO: 1903690~negative regulation of wound healing, spreading of epidermal cells	GO: 0051893~regulation of focal adhesion assembly
GO: 0035264~multicellular organism growth	GO: 0006376~mRNA splice site selection
GO: 0006661~phosphatidylinositol biosynthetic process	GO: 2000785~regulation of autophagosome assembly
GO: 0050821~protein stabilization	GO: 0006921~cellular component disassembly involved in execution phase of apoptosis
GO: 0001889~liver development	GO: 0016055~Wnt signaling pathway
GO: 0032147~activation of protein kinase activity	GO: 0001701~in utero embryonic development
GO: 0048589~developmental growth	GO: 0019886~antigen processing and presentation of exogenous peptide antigen via MHC class II
GO: 0031054~pre-miRNA processing	GO: 0010761~fibroblast migration
GO: 0007010~cytoskeleton organization	GO: 0048280~vesicle fusion with Golgi apparatus
GO: 0000722~telomere maintenance via recombination	GO: 0008543~fibroblast growth factor receptor signaling pathway

GO: 0007032~endosome organization	GO: 0007052~mitotic spindle organization
GO: 0018026~peptidyl-lysine monomethylation	GO: 0051056~regulation of small GTPase mediated signal transduction
GO: 0072091~regulation of stem cell proliferation	GO: 0016477~cell migration
GO: 0035196~production of miRNAs involved in gene silencing by miRNA	GO: 0006606~protein import into nucleus
GO: 0046825~regulation of protein export from nucleus	GO: 0010501~RNA secondary structure unwinding
GO: 0000288~nuclear-transcribed mRNA catabolic process, deadenylation-dependent decay	GO: 0008356~asymmetric cell division
GO: 0007183~SMAD protein complex assembly	GO: 0071044~histone mRNA catabolic process
GO: 0007223~Wnt signaling pathway, calcium modulating pathway	GO: 0043923~positive regulation by host of viral transcription
GO: 0071897~DNA biosynthetic process	GO: 0007064~mitotic sister chromatid cohesion
GO: 0006259~DNA metabolic process	GO: 0016578~histone deubiquitination
GO: 0048511~rhythmic process	GO: 1901673~regulation of mitotic spindle assembly
GO: 0007179~transforming growth factor beta receptor signaling pathway	GO: 0006509~membrane protein ectodomain proteolysis
GO: 1903507~negative regulation of nucleic acid-templated transcription	GO: 0003183~mitral valve morphogenesis

GO: 0017015~regulation of transforming growth factor beta receptor signaling pathway	GO: 0035194~posttranscriptional gene silencing by RNA
GO: 0021591~ventricular system development	GO: 0042752~regulation of circadian rhythm
GO: 1901224~positive regulation of NIK/NF-kappaB signaling	GO: 0010971~positive regulation of G2/M transition of mitotic cell cycle
GO: 0043923~positive regulation by host of viral transcription	GO: 0071539~protein localization to centrosome
GO: 0006977~DNA damage response, signal transduction by p53 class mediator resulting in cell cycle arrest	GO: 0007020~microtubule nucleation
GO: 0045815~positive regulation of gene expression, epigenetic	GO: 0061512~protein localization to cilium
GO: 0000902~cell morphogenesis	GO: 0043967~histone H4 acetylation
	GO: 1990090~cellular response to nerve growth factor stimulus
	GO: 0008542~visual learning
	GO: 0031297~replication fork processing
	GO: 0035195~gene silencing by miRNA
	GO: 0016601~Rac protein signal transduction
	GO: 0006895~Golgi to endosome transport
	GO: 0034389~lipid particle organization
	GO: 0080182~histone H3-K4 trimethylation
	GO: 0043123~positive regulation of I-kappaB kinase/NF-kappaB signaling

	GO: 0016197~endosomal transport
	GO: 0032467~positive regulation of cytokinesis
	GO: 0043547~positive regulation of GTPase activity
	GO: 0001843~neural tube closure
	GO: 0051291~protein heterooligomerization
	GO: 0006370~7-methylguanosine mRNA capping
	GO: 0051297~centrosome organization
	GO: 0006977~DNA damage response, signal transduction by p53 class mediator resulting in cell cycle arrest
	GO: 0000902~cell morphogenesis
	GO: 0007050~cell cycle arrest
	GO: 0006610~ribosomal protein import into nucleus
	GO: 0007183~SMAD protein complex assembly
	GO: 0010470~regulation of gastrulation
	GO: 0046825~regulation of protein export from nucleus
	GO: 2000643~positive regulation of early endosome to late endosome transport

	GO: 0033683~nucleotide-excision repair, DNA incision
	GO: 0007094~mitotic spindle assembly checkpoint
	GO: 0072583~clathrin-mediated endocytosis
	GO: 0017015~regulation of transforming growth factor beta receptor signaling pathway
	GO: 1902895~positive regulation of pri-miRNA transcription from RNA polymerase II promoter
	GO: 0000042~protein targeting to Golgi
	GO: 0035335~peptidyl-tyrosine dephosphorylation
	GO: 0009791~post-embryonic development
	GO: 0043484~regulation of RNA splicing
	GO: 0030518~intracellular steroid hormone receptor signaling pathway
	GO: 0000712~resolution of meiotic recombination intermediates
	GO: 0070507~regulation of microtubule cytoskeleton organization
	GO: 0006349~regulation of gene expression by genetic imprinting
	GO: 0010608~posttranscriptional regulation of gene expression
	GO: 0051642~centrosome localization

	GO: 1904355~positive regulation of telomere capping
	GO: 0042073~intraciliary transport
	GO: 1903830~magnesium ion transmembrane transport
	GO: 0061014~positive regulation of mRNA catabolic process
	GO: 0090162~establishment of epithelial cell polarity
	GO: 0035278~miRNA mediated inhibition of translation
	GO: 0070816~phosphorylation of RNA polymerase II C-terminal domain
	GO: 0010032~meiotic chromosome condensation
	GO: 0070201~regulation of establishment of protein localization
	GO: 0046580~negative regulation of Ras protein signal transduction
	GO: 0007223~Wnt signaling pathway, calcium modulating pathway
	GO: 0007224~smoothened signaling pathway
	GO: 0061025~membrane fusion
	GO: 0034644~cellular response to UV
	GO: 0016241~regulation of macroautophagy

	GO: 0030512~negative regulation of transforming growth factor beta receptor signaling pathway
	GO: 0036092~phosphatidylinositol-3-phosphate biosynthetic process
	GO: 0045773~positive regulation of axon extension
	GO: 0071480~cellular response to gamma radiation
	GO: 0007249~I-kappaB kinase/NF-kappaB signaling
	GO: 0048568~embryonic organ development
	GO: 0016571~histone methylation
	GO: 0001702~gastrulation with mouth forming second
	GO: 0007276~gamete generation
	GO: 0008089~anterograde axonal transport
	GO: 0006265~DNA topological change
	GO: 0010224~response to UV-B
	GO: 0042921~glucocorticoid receptor signaling pathway
	GO: 0032886~regulation of microtubule-based process
	GO: 0048096~chromatin-mediated maintenance of transcription

	GO: 0035562~negative regulation of chromatin binding
	GO: 0060039~pericardium development
	GO: 0048489~synaptic vesicle transport
	GO: 0051457~maintenance of protein location in nucleus
	GO: 0030516~regulation of axon extension
	GO: 0043488~regulation of mRNA stability
	GO: 0042771~intrinsic apoptotic signaling pathway in response to DNA damage by p53 class mediator
	GO: 0030100~regulation of endocytosis
	GO: 0042059~negative regulation of epidermal growth factor receptor signaling pathway
	GO: 0046329~negative regulation of JNK cascade
	GO: 0030900~forebrain development
	GO: 0035556~intracellular signal transduction
	GO: 0006310~DNA recombination
	GO: 0007264~small GTPase mediated signal transduction
	GO: 0045022~early endosome to late endosome transport
	GO: 0030705~cytoskeleton-dependent intracellular transport

	GO: 0032922~circadian regulation of gene expression
	GO: 0006461~protein complex assembly
	GO: 0032212~positive regulation of telomere maintenance via telomerase
	GO: 0070830~bicellular tight junction assembly
	GO: 0006296~nucleotide-excision repair, DNA incision, 5'-to lesion
	GO: 0048813~dendrite morphogenesis
	GO: 0006914~autophagy

Supplemental table S4 KEGG pathways of GPNCA and GSK3B in LIHC related to Supplemental Figure S4F

LIHC_GPNCA_KEGG	LIHC_GSK3B_KEGG
hsa03460: Fanconi anemia pathway	hsa04144: Endocytosis
hsa03040: Spliceosome	hsa04120: Ubiquitin mediated proteolysis
hsa04120: Ubiquitin mediated proteolysis	hsa03460: Fanconi anemia pathway
hsa03015: mRNA surveillance pathway	hsa03040: Spliceosome
hsa03013: RNA transport	hsa03015: mRNA surveillance pathway
hsa03018: RNA degradation	hsa04722: Neurotrophin signaling pathway
hsa04110: Cell cycle	hsa03013: RNA transport
hsa04722: Neurotrophin signaling pathway	hsa04012: ErbB signaling pathway

hsa04520: Adherens junction	hsa04550: Signaling pathways regulating pluripotency of stem cells
hsa04068: FoxO signaling pathway	hsa04110: Cell cycle
hsa05203: Viral carcinogenesis	hsa05211: Renal cell carcinoma
hsa00310: Lysine degradation	hsa04520: Adherens junction
hsa05210: Colorectal cancer	hsa05210: Colorectal cancer
hsa05211: Renal cell carcinoma	hsa03018: RNA degradation
hsa04144: Endocytosis	hsa04070: Phosphatidylinositol signaling system
hsa04350: TGF-beta signaling pathway	hsa04390: Hippo signaling pathway
hsa05168: Herpes simplex infection	hsa00562: Inositol phosphate metabolism
hsa04919: Thyroid hormone signaling pathway	hsa05213: Endometrial cancer
hsa04070: Phosphatidylinositol signaling system	hsa04919: Thyroid hormone signaling pathway
hsa00562: Inositol phosphate metabolism	hsa04810: Regulation of actin cytoskeleton
hsa04550: Signaling pathways regulating pluripotency of stem cells	hsa05161: Hepatitis B
hsa05212: Pancreatic cancer	hsa04114: Oocyte meiosis
hsa04012: ErbB signaling pathway	hsa04912: GnRH signaling pathway
hsa04914: Progesterone-mediated oocyte maturation	hsa05200: Pathways in cancer
hsa05213: Endometrial cancer	hsa04071: Sphingolipid signaling pathway
hsa04114: Oocyte meiosis	hsa05212: Pancreatic cancer

hsa03440: Homologous recombination	hsa03022: Basal transcription factors
hsa05161: Hepatitis B	hsa05168: Herpes simplex infection
hsa05166: HTLV-I infection	hsa04350: TGF-beta signaling pathway
hsa04910: Insulin signaling pathway	hsa05203: Viral carcinogenesis
hsa04310: Wnt signaling pathway	hsa04910: Insulin signaling pathway
hsa05215: Prostate cancer	hsa04660: T cell receptor signaling pathway
hsa04010: MAPK signaling pathway	hsa04720: Long-term potentiation
hsa05231: Choline metabolism in cancer	hsa04014: Ras signaling pathway
hsa04912: GnRH signaling pathway	hsa05205: Proteoglycans in cancer
hsa04917: Prolactin signaling pathway	hsa03430: Mismatch repair
hsa05220: Chronic myeloid leukemia	hsa05215: Prostate cancer
hsa04340: Hedgehog signaling pathway	hsa04668: TNF signaling pathway
hsa03022: Basal transcription factors	hsa04962: Vasopressin-regulated water reabsorption
hsa04668: TNF signaling pathway	hsa05223: Non-small cell lung cancer
hsa05223: Non-small cell lung cancer	hsa04510: Focal adhesion
hsa03420: Nucleotide excision repair	hsa04010: MAPK signaling pathway
hsa04660: T cell receptor signaling pathway	hsa04064: NF-kappa B signaling pathway
hsa04662: B cell receptor signaling pathway	hsa00310: Lysine degradation
hsa04210: Apoptosis	hsa05131: Shigellosis
hsa04390: Hippo signaling pathway	hsa03420: Nucleotide excision repair
hsa05152: Tuberculosis	hsa04310: Wnt signaling pathway

	hsa04622: RIG-I-like receptor signaling pathway
	hsa05231: Choline metabolism in cancer
	hsa05160: Hepatitis C
	hsa04917: Prolactin signaling pathway
	hsa04068: FoxO signaling pathway
	hsa04728: Dopaminergic synapse
	hsa05220: Chronic myeloid leukemia
	hsa04210: Apoptosis
	hsa05214: Glioma
	hsa00563: Glycosylphosphatidylinositol(GPI)-anchor biosynthesis
	hsa05100: Bacterial invasion of epithelial cells
	hsa05152: Tuberculosis
	hsa04330: Notch signaling pathway
	hsa05166: HTLV-I infection
	hsa05120: Epithelial cell signaling in Helicobacter pylori infection
	hsa04914: Progesterone-mediated oocyte maturation
	hsa04916: Melanogenesis
	hsa05202: Transcriptional misregulation in cancer

	hsa04662: B cell receptor signaling pathway
	hsa04340: Hedgehog signaling pathway
